# Design and Simulation Study of a Jumping Takeoff Mechanism Inspired by the Hindleg Kinematics of Asian Migratory Locust, *Locusta migratoria*

**DOI:** 10.3390/biomimetics11090627

**Published:** 2026-09-02

**Authors:** Yuhang Wang, Yaohui Wang, Wenshan Wang, Huan Shen, Eize J. Stamhuis, Lining Sun, Qian Wang, Chao Liu

**Affiliations:** 1 Robotics and Microsystems Center, College of Mechanical and Electrical Engineering, Soochow University, Suzhou 215021, China; 20254229009@stu.suda.edu.cn (Y.W.); 2430412062@stu.suda.edu.cn (Y.W.); 2362405069@stu.suda.edu.cn (W.W.); lnsun@suda.edu.cn (L.S.); 2 College of Mechanical and Electrical Engineering, Nanjing University of Aeronautics and Astronautics, Nanjing 210016, China; shenhuan99@nuaa.edu.cn; 3 Experimental Zoology Group, Wageningen University, 6708 PB Wageningen, The Netherlands; 4 Faculty of Science and Engineering, University of Groningen, 9747 AG Groningen, The Netherlands; e.j.stamhuis@rug.nl

**Keywords:** bio-inspired robotics, locust-inspired, jumping takeoff mechanism, aerodynamics

## Abstract

The legs of flying insects play a critical role in enabling seamless transitions between aerial and terrestrial environments. These appendages serve multiple functions, including landing, walking, jumping, and transitioning from jumping to flight (takeoff). Such capabilities have inspired engineers to seek similar multimodal mechanisms in Flapping-Wing Aerial Robots (FWARs) to expand their operational versatility across diverse environments. However, designing multimodal mechanisms with distinct kinematic and propulsive characteristics remains challenging, particularly in the domain of autonomous jump takeoff for FWARs, where research remains relatively sparse. In this study, inspired by the jumping takeoff strategy and hindleg kinematics of the Asian migratory locust (*Locusta migratoria*), we propose a functional bio-inspired jumping takeoff mechanism that extracts selected mechanical principles of the locust jumping system, including elastic energy accumulation, temporary mechanical locking, and rapid energy release. The mechanism employs a gear–crank–slider transmission system and utilizes one-way bearings to regulate the locking and disengaging states, enabling the storage and rapid release of energy for jump takeoff, thereby achieving autonomous takeoff of the robot. Adams dynamic simulations show that at a torsion spring angle of 40°, the mechanism achieves a maximum resultant velocity of 1.955 m/s, a jump height of 168.2 mm, and a horizontal displacement upon landing of 134.6 mm. Ansys Fluent (2024 R2) simulations under multiple operating conditions further confirm that the aerodynamic performance is optimal at a takeoff angle of attack(*α*) of 5° with a torsion spring angle(*β*) of 40°, yielding a lift-to-drag ratio of 3.005. This work presents a functional bio-inspired jumping takeoff mechanism based on selected mechanical principles of locust jumping, providing a potential approach for improving the autonomous takeoff capability of small-scale FWARs.

## 1. Introduction

Insect locomotion strategies [1] are products of natural selection, integrating morphological structures, physiological functions, and behavioral patterns to achieve effective displacement within their environments [2]. In many insects, such as locusts [3], fleas [4], beetles [4], and ladybugs [1], saltatorial locomotion in the form of jumping or jumping takeoff serves as an effective means of overcoming obstacles [5,6,7,8]. These examples demonstrate that jump-assisted takeoff is a highly convergent and remarkably effective locomotion strategy among small insects [1,9]. Its core advantages [1,9] include execution on a millisecond timescale, enabling the animal to surpass the perceptual and neural response thresholds of most natural predators (rapidness). Through a “catapult mechanism” involving pre-storage and rapid release of energy, the system achieves peak power outputs far exceeding the limits of direct muscle contraction, thereby generating extremely high initial acceleration (high power output). This strategy [1] serves multiple functions in survival activities: it can act as a critical escape or defensive behavior, be used in ambush predation, or serve as an initial power source for efficient takeoff in flying-capable organisms (functional polymorphism). When traversing complex ground micro-terrains—such as dense vegetation, ground fissures, and uneven surfaces—jumping [1] provides an efficient solution for rapidly overcoming local obstacles (terrain adaptability). These diverse biological prototypes have provided rich sources of inspiration for the design of bio-inspired jumping robots, particularly in elastic energy-storage strategies, mechanical latch–release mechanisms, and multi-segment coordination during leg extension and body launching.

**Figure 1:**
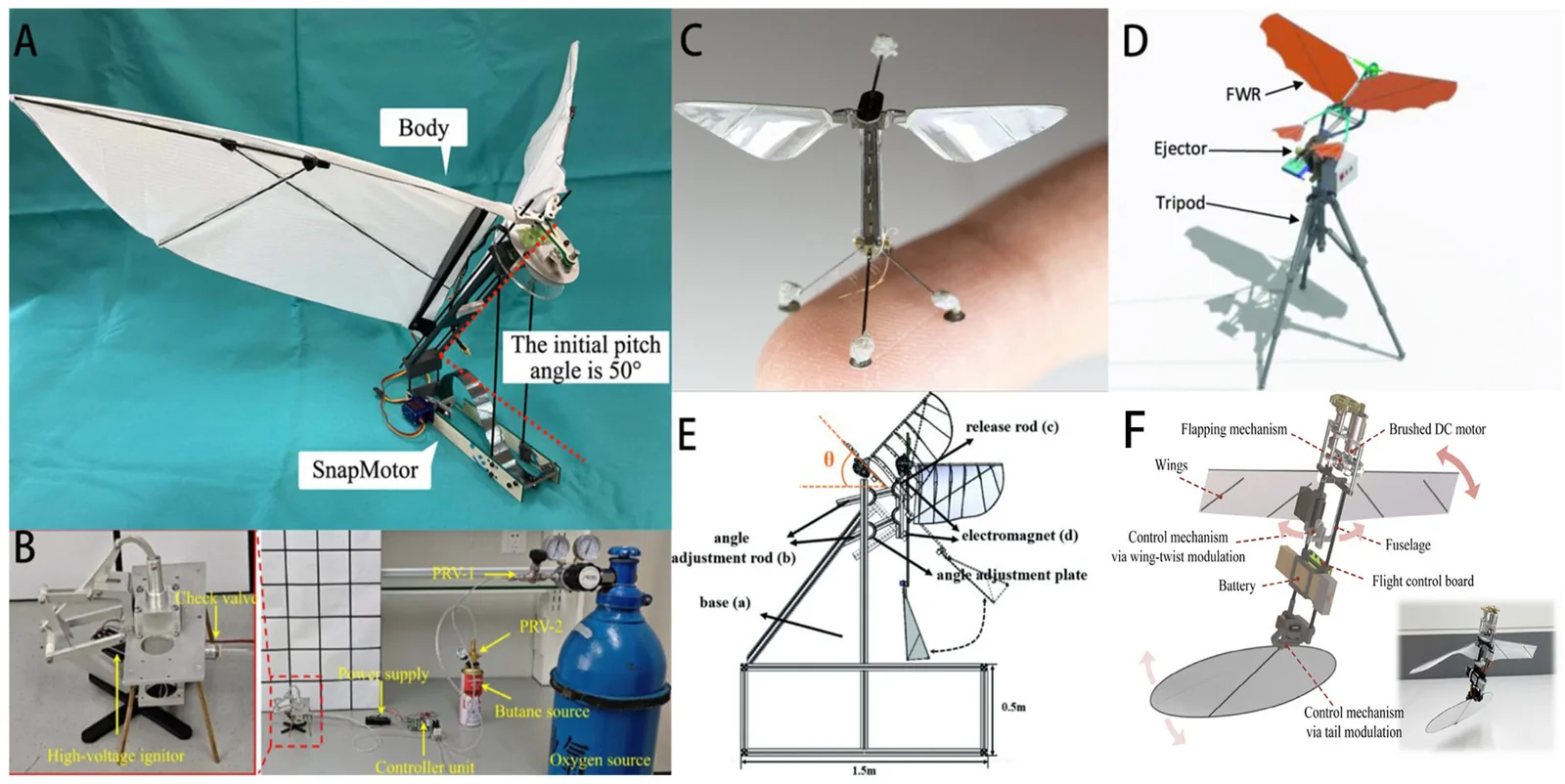
Examples of existing flapping-wing robots with jumping mechanisms. (**A**) Ornibot; (**B**) Gal Ribak’s insect-inspired jumping robot; (**C**) Robo Bee; (**D**) slider-crank ejecting system with FWAR; (**E**) osprey-inspired eagle-scale FWAR; (**F**) two-wing flapping-wing micro aerial vehicle (MAV).

Researchers have integrated jumping mechanisms into flapping-wing robots (Figure 1A) to achieve jump-assisted takeoff. This strategy can effectively prevent wing–ground collisions, reduce the pitching moment during the initial takeoff phase, and improve attitude stability [10]. Several studies have reviewed the stability and controllability of insect-inspired jumping robots. Although power amplification mechanisms can overcome the limited power density of small-scale actuators, these systems still inherit the limitations of insect jumping, such as restricted jumping capability [11]. Inspired by flea beetles (Figure 1B), some researchers developed a jumping robot powered by a butane–oxygen combustion explosion propulsion system. This method utilizes the high energy density of chemical fuels to achieve instantaneous high-power output. The prototype reached a jump height of 20 cm (1.67 times its body length). However, issues including continuous fuel supply, impact resistance, and weight reduction remain challenging [12]. For instance, Harvard University’s Robo Bee (Figure 1C) achieved takeoff and hovering in a millimeter-scale flapping-wing aerial robots (FWARs) using piezoelectric actuators, but it relies on external power and lacks autonomous takeoff capability. Although recent studies have developed bio-inspired landing mechanisms for it, takeoff still requires human intervention [13]. Researchers have designed an ejecting system based on a slider-crank mechanism (Figure 1D) to address the autonomous takeoff problem of flapping-wing robots. This system can provide a takeoff velocity of 4 m/s for a 270 g flapping-wing robot with only a 3.2 g additional payload and is suitable for rough terrain; however, it can only provide a single assisted takeoff [14]. The Highly Dragon bio-inspired dragonfly employs four independently controllable resonant direct-drive flapping mechanisms in a compact design, but suffers from complex system architecture and high energy consumption, and cannot achieve autonomous takeoff [15]. Inspired by the takeoff process of ospreys, one research team proposed a bio-inspired takeoff strategy (Figure 1E) and established a corresponding dynamic model and self-takeoff condition analysis. This study achieved the first ground self-takeoff of an eagle-scale flapping-wing robot, providing a viable approach for autonomous takeoff of large-scale flapping-wing robots; however, small-scale flapping-wing robots still lack dedicated autonomous takeoff mechanisms [16]. A bio-inspired two-winged flapping-wing micro air vehicle with a gear-based flapping mechanism (Figure 1F). The prototype achieved vertical takeoff and hover flight, demonstrating the potential of mechanical optimization and aerodynamic design for improving the performance of FW-MAVs [17].

Significant progress has been made in research on jumping mechanisms for bio-inspired flapping-wing flying robots, particularly in mechanism innovation (e.g., multi-link bio-inspired legs, energy latch release mechanisms), actuation methods (e.g., motor-spring, combustion explosion, hydraulic/pneumatic), and exploration of biological principles (catapult-based vs. non-catapult-based) [18,19,20]. A comparison of representative FWARs with jumping-assisted or takeoff mechanisms is summarized in Table 1, highlighting the differences in takeoff strategies, performance, reusability, autonomous capability, and system complexity. Nevertheless, most robots still face challenges such as insufficient jumping capability, inability to achieve autonomous control, or dependence on specific takeoff conditions, failing to replicate the efficient and repeatable jumping locomotion observed in insects [21,22,23,24,25].

Although previous studies have investigated locust hindleg biomechanics, elastic energy-storage mechanisms, spring-actuated jumping systems, takeoff stability, and jumping trajectory characteristics, these aspects have generally been addressed separately. Existing locust-inspired robotic studies have primarily focused on reproducing jumping kinematics, improving elastic energy storage and release, or enhancing jumping stability, whereas relatively limited attention has been paid to the coordinated consideration of biological jumping characteristics, controllable mechanical energy storage–release, and aerodynamic performance during the jumping takeoff phase of small-scale FWARs. In particular, how experimentally observed locust jumping characteristics can be translated into functional engineering requirements and further coupled with the mechanical and aerodynamic design of an autonomous takeoff system remains insufficiently explored. Therefore, an integrated design framework linking biological observation, jumping mechanism development, dynamic performance evaluation, and aerodynamic analysis is still required for autonomous takeoff of small-scale FWARs.

To address this research gap, this study proposes a functional bio-inspired jumping takeoff mechanism for small-scale FWARs based on the jumping takeoff characteristics of the Asian migratory locust (*Locusta migratoria*). First, high-speed motion capture and trajectory analysis are conducted to investigate locust jumping under different ground-slope conditions and to extract functional characteristics relevant to robotic takeoff, including takeoff timescale, coordinated hindleg extension, ground-contact stability, and characteristic velocity range. Based on these observations, a controllable jumping mechanism integrating elastic energy storage, mechanical locking, and rapid energy release is developed. Its dynamic performance is subsequently evaluated using Adams 2025, and Ansys Fluent (2024 R2) is further employed to investigate the aerodynamic characteristics under different takeoff configurations. Unlike studies aimed at directly reproducing the anatomical or physiological structure of locust hindlegs, the present work focuses on translating selected functional principles of locust jumping into an engineering takeoff mechanism and integrating biological observation, mechanical design, dynamic simulation, and aerodynamic evaluation within a unified framework. The study is expected to provide a feasible approach for improving the autonomous takeoff capability of small-scale FWARs.

## 2. Biological Inspiration from Locust Jumping Takeoff

### 2.1. Locust Specimens

**Figure 2:**
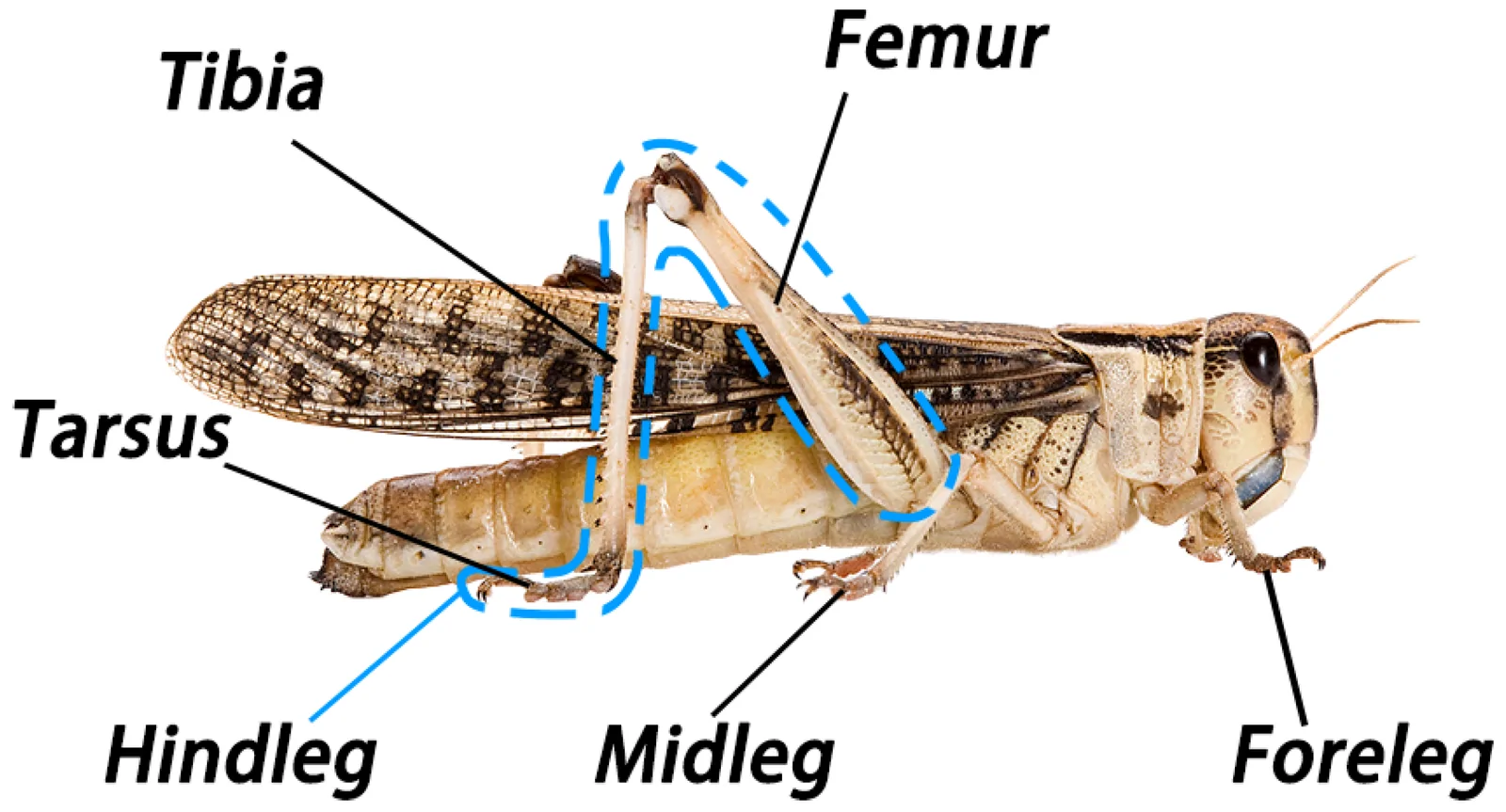
Leg morphology of the adult Asian migratory locust (*Locusta migratoria*).

The specimens used in this study were the Asian migratory locust (*Locusta migratoria*), belonging to the family Acrididae and order Orthoptera, collected from Xishuangbanna, Yunnan Province, China (Figure 2). Twenty-five adult male individuals were selected, with body lengths ranging from 32.4 to 48.1 mm (mean 37.7 mm ± 0.2 mm) and body widths approximately between 8.0 and 9.5 mm. Their body coloration is predominantly grass-green or yellowish-brown. The adult thorax bears three pairs of legs: the forelegs and midlegs are relatively short and primarily used for crawling and support, whereas the hindlegs are extremely robust and specialized for jumping, with particularly well-developed and thickened femora and slender tibiae bearing a brownish hue, well adapted for leaping. All leg samples used for experimental measurements were obtained from freshly anesthetized specimens and were handled in strict compliance with established animal ethical standards.

### 2.2. Motion Capture of Locust Jumping Takeoff

**Figure 3:**
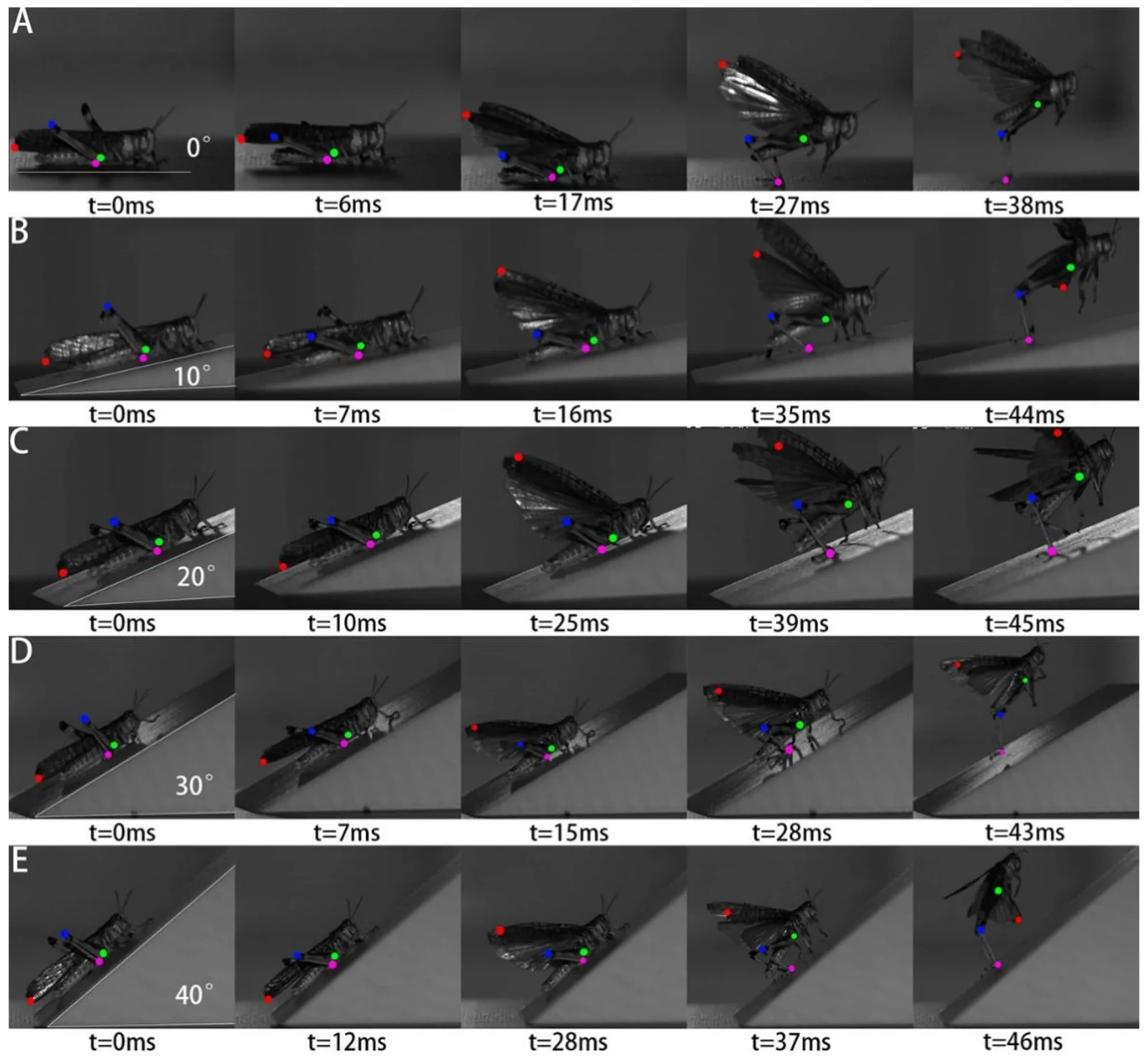
High-speed camera motion capture of locust jumping takeoff. (**A**) Horizontal surface jumping takeoff; (**B**) jumping takeoff on a 10° slope; (**C**) jumping takeoff on a 20° slope; (**D**) jumping takeoff on a 30° slope; (**E**) jumping takeoff on a 40° slope. The colored dots indicate the tracked body landmarks used for kinematic analysis, including the wing tip (red), femur tip (green), tibia tip (blue), and tarsus tip (magenta).

As shown in Figure 3, the jumping takeoff of locusts is a highly efficient locomotor mode optimized through natural selection, involving energy storage and rapid release. Investigating this jumping process is of great significance for the design of the takeoff mechanism. Observations using a high-speed camera (Revealer-M230M/C Pro, Hefei, China) revealed that the jumping takeoff consists of three consecutive phases. Energy storage phase (Figure 3A, t = 0–6 ms): The locust lowers its body and folds the femur and tibia into a compact “Z”-shaped posture. During jump preparation, contraction of the extensor system progressively loads the elastic structures of the hindlegs. According to Bennet-Clark, the strain energy required for jumping is distributed between the semi-lunar processes and the extensor tibiae apodemes rather than being stored exclusively in a resilin-based structure [26]. Locking phase (Figure 3A, 6–17 ms): A mechanical interlocking mechanism between skeletal components locks the energy-storing configuration to prevent premature energy release. Subsequently, muscles continue to contract, gradually reducing the interlocking force between skeletal components in preparation for energy release. Jumping takeoff phase (Figure 3A, 17–38 ms): Neural signals trigger the jumping switch, activating the fast extensor muscles and causing the instantaneous release of stored energy. This process is extremely brief—from energy storage to jumping takeoff, the locust requires only 0 to 50 ms. The interlocking structures between the hindleg segments are released, and the tibiae instantly spring open, propelling the body in a catapult-like motion, after which the wings are opened to complete takeoff. The ground reaction force of the hindlegs can reach up to 20 times the locust’s body weight. The leg acceleration reaches as high as 20 m/s^2^, and the instantaneous velocity can reach 3 m/s (based on 50 jumps, ±0.4 error), which is several tens of times the locust’s body length.

### 2.3. Kinematics of Locust Jumping Takeoff

The kinematic parameters reported below were obtained from repeated jumping trials. Because biological jumping exhibits inter-individual and inter-trial variability, the experimental data are reported as mean values together with their corresponding variability where available. The analysis therefore focuses primarily on the overall variation trends across slope conditions rather than treating individual values as deterministic design parameters.

**Figure 4:**
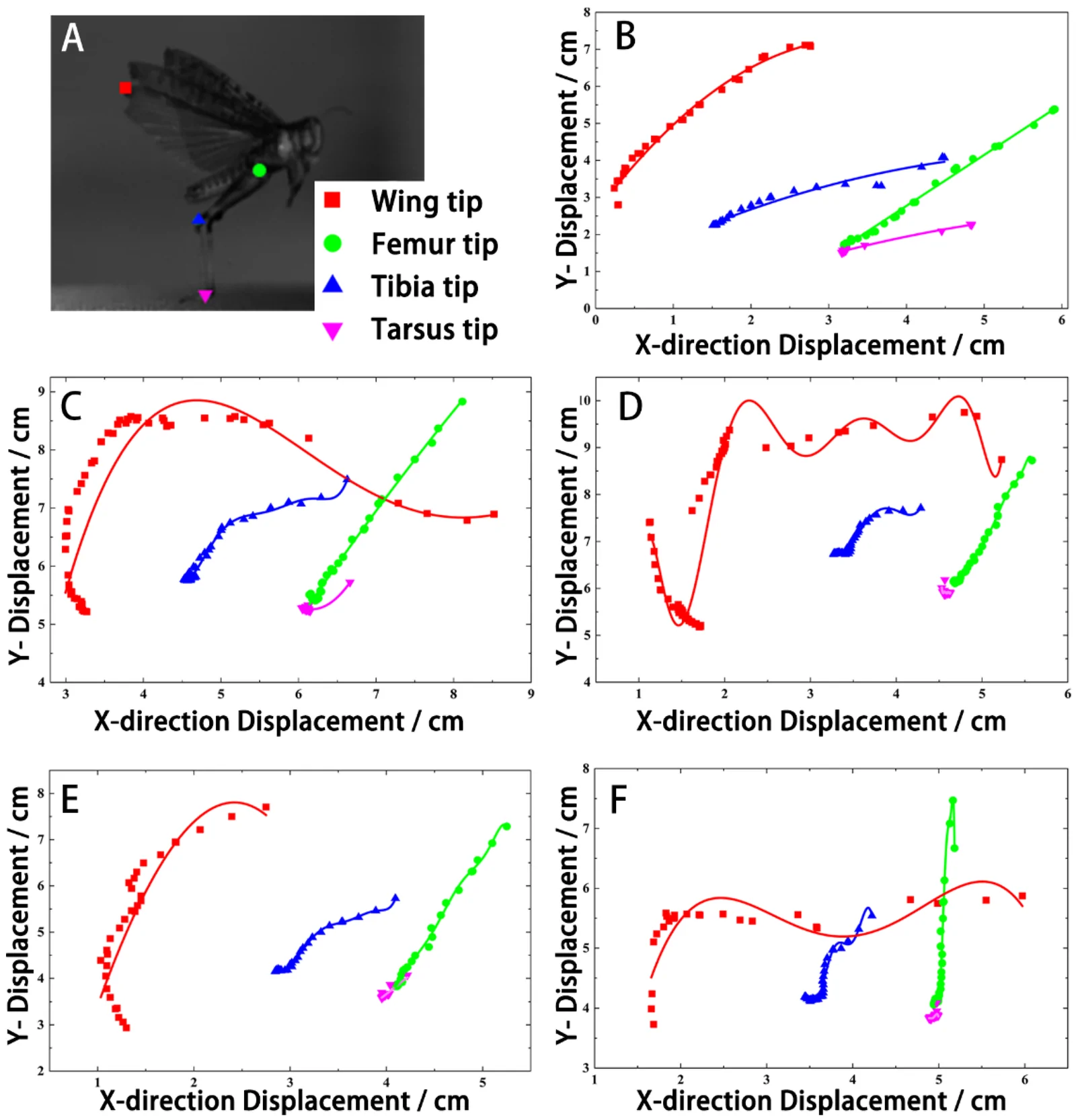
Trajectory analysis of locust jumping takeoff on inclined surfaces of different slopes. (**A**) Schematic diagram of the markers on the locust’s body parts for trajectory analysis; (**B**) trajectory analysis of locust takeoff on a horizontal surface (0°); (**C**) trajectory analysis of locust takeoff on a 10° slope; (**D**) trajectory analysis of locust takeoff on a 20° slope; (**E**) trajectory analysis of locust takeoff on a 30° slope; (**F**) trajectory analysis of locust takeoff on a 40° slope.

The specific marker locations are shown in Figure 4A. As shown in Figure 4B, on a horizontal surface, the displacement curve of the locust’s tarsus increases smoothly, reaching a peak of approximately 7.1 mm. Prior to takeoff, the tarsus pushes steadily backward and downward against the ground without noticeable sliding. The vertical velocity of the tibia reaches approximately 1.25 m/s, while the horizontal velocity is about 0.85 m/s, with the vertical component being clearly dominant. The velocities of the femur and tibia are essentially synchronized at the moment of takeoff, indicating good coordination between the extension of the femur and tibia. The entire jumping trajectory approximates a symmetric parabola, with the locust’s body lifting off almost vertically, demonstrating that on a horizontal surface it can achieve a stable vertical takeoff with minimal energy loss.

As shown in Figure 4C, when the surface is inclined at 10°, the tarsal displacement increases to approximately 7.5 mm, requiring the locust to adopt a larger thrust amplitude to obtain sufficient vertical lift. The vertical velocity of the tibia increases to 1.45 m/s, and the horizontal velocity rises to 1.05 m/s, slightly reducing the vertical dominance. Compared with the 0° case, the extension phases of the femur and tibia remain consistent, but the increment in horizontal velocity is more pronounced, indicating that the locust begins to utilize the horizontal component of the slope to assist forward propulsion. After takeoff, the body tilts slightly forward, and the trajectory shifts slightly upward and forward, remaining overall stable.

As shown in Figure 4D, when the slope reaches 20°, the tarsal displacement further increases to 8.1 mm, requiring the hindlegs to extend more fully forward. The vertical velocity of the tibia reaches 1.65 m/s, and the horizontal velocity reaches 1.35 m/s, with their ratio dropping to approximately 1.2, weakening the vertical dominance. The extension amplitude of the knee joint (between the femur and tibia) increases markedly, and the peak velocities of both segments occur almost simultaneously, indicating that the locust maintains good lower-limb coordination even on steeper slopes. The takeoff angle is approximately 65°, with the body tilting forward noticeably, beginning to exhibit a compound “jump-and-thrust” locomotion mode.

As shown in Figure 4E, on a 30° slope, the tarsal displacement reaches its maximum among all tested angles, at approximately 8.6 mm, indicating that the locust fully utilizes the extension range of the hindlegs to overcome the downslope component of gravity. The vertical velocity of the tibia increases to 1.85 m/s, and the horizontal velocity reaches 1.65 m/s, with a ratio close to 1.1 and a takeoff angle of approximately 50°. Notably, the velocity curves of the femur and tibia almost completely overlap, indicating that the knee and ankle joints achieve optimal synchronization at the moment of maximum extension, suggesting that 30° is the slope at which the locust exhibits the best locomotor coordination. No slipping or attitude oscillation occurs throughout the jumping process.

As shown in Figure 4F, when the slope increases to 40°, the tarsal displacement decreases to approximately 7.9 mm, and a brief reversal of horizontal velocity (slight backward sliding) occurs within milliseconds before takeoff, indicating that the excessively steep slope prevents the tarsus from securely gripping the ground. The vertical velocity of the tibia continues to rise to 2.05 m/s, and the horizontal velocity reaches 1.95 m/s, with a ratio close to 1.0; however, the velocity curves exhibit noticeable fluctuations and reduced coordination. Although the locust can still complete takeoff and propel itself forward, stability deteriorates significantly, with the body tilting forward to nearly 45° and a longer forward displacement upon landing. This indicates that 40° approaches the limit of the locust’s jumping takeoff capability.

In summary, the jumping trajectories across the five slopes reveal that slope angle significantly influences the locust’s takeoff strategy. As the slope increases from 0° to 30°, the tarsal displacement increases monotonically from 7.1 mm to 8.6 mm, and both vertical and horizontal velocities of the tibia continue to rise; however, the rate of increase in horizontal velocity is notably faster than that of vertical velocity, causing the takeoff angle to decrease gradually from near 90° to approximately 50°. This indicates that on steeper slopes, the locust actively adjusts its energy allocation strategy—shifting from a primarily vertical takeoff focused on overcoming gravity to an oblique jump that balances vertical ascent with horizontal propulsion. In terms of locomotor coordination, the femur and tibia exhibit good velocity synchronization across the 0–20° range, while at 30° the velocity curves of the two segments almost completely overlap, suggesting that the knee and ankle joints achieve the most coordinated extension at this angle, making 30° the optimal slope for locust jumping takeoff. However, when the slope increases to 40°, the tarsal displacement decreases, brief backward sliding occurs prior to takeoff, and velocity fluctuations intensify, indicating that the excessively steep surface approaches the limit of the tarsus’s gripping capacity. Although takeoff can still be completed, stability and controllability are significantly compromised. Overall, the locust demonstrates good terrain adaptability within the tested 0–30° range. Among the tested conditions, the 30° slope exhibited the highest degree of femur–tibia synchronization, whereas the increased sliding and velocity fluctuations observed at 40° indicate reduced jumping stability under the steepest tested condition. The inclined-surface experiments were not intended to reproduce the complete ecological jumping behaviors of locusts, but rather to analyze how different ground-contact conditions influence hindleg coordination and takeoff performance, providing design references for robots operating on complex terrains. These observations indicate that the high-performance jumping ability of locusts does not originate from muscle contraction alone, but from the coordinated interaction between muscle actuation, elastic energy storage structures, and mechanical locking mechanisms. Therefore, the biological inspiration extracted in this study is mainly the functional strategy of “slow energy accumulation–temporary storage–rapid release” rather than the direct replication of specific biological tissues.

## 3. Design of the Jumping Takeoff Mechanism Inspired by Locust HindLeg Kinematics

### 3.1. Biomimetic Design Feature Extraction 

Based on the motion capture and trajectory analysis of locust jumping takeoff, which combines both jumping and flying capabilities, the following bio-inspired elements were extracted:

(1) Tarsal claw configuration design: Inspired by the synergistic ground-engaging and locking mechanism of the tarsal claws and adhesive pads on the locust hindlegs, the robotic foot structure was optimized by modifying the orientation of the tarsal segments. The claw-like structures are designed to interlock with microscopic surface irregularities, thereby increasing the static friction coefficient and effectively suppressing body sliding during the thrust phase of takeoff.

(2) Center of mass layout optimization: By integrally arranging the drive motor, transmission components, and energy storage elements, the pitching moment generated at the instant of ground push-off is reduced.

(3) Bilateral jumping mechanism arrangement: Based on the synchronous jumping motion of the locust’s paired hindlegs, two sets of jumping mechanisms are symmetrically arranged to balance radial forces on the body and prevent lateral instability during takeoff.

Thus, the biological experiments were not used to directly determine the detailed geometry or spring parameters of the robotic mechanism. Instead, they provided functional targets for takeoff timescale, hindleg coordination, ground-contact stability, and velocity magnitude, while the specific mechanical parameters were subsequently determined through engineering design and dynamic simulation.

### 3.2. Design of the Bio-Inspired Jumping Takeoff Mechanism

**Figure 5:**
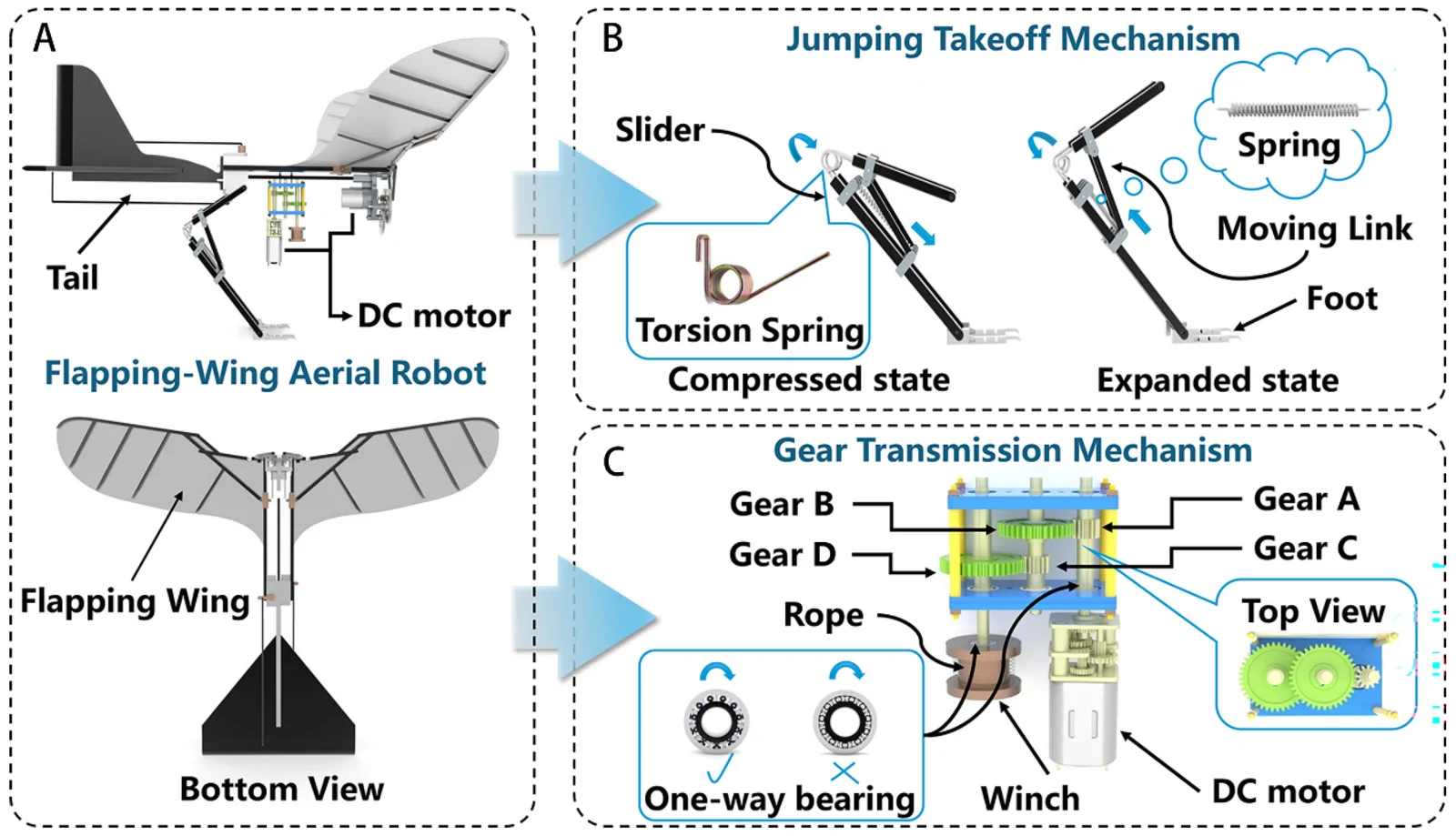
Design of the locust-inspired jumping takeoff mechanism. (**A**) Flapping-wing robot integrated with the locust-inspired mechanism; (**B**) leg mechanism; (**C**) gear transmission mechanism.

In this study, a bio-inspired locust jumping takeoff mechanism was designed. Figure 5A presents the assembly drawing of the flapping-wing robot integrated with the locust-inspired mechanism, which includes the flapping wing, tail, drive motor, and other components, with a bottom view provided to illustrate the underside structural layout. Figure 5B shows the leg mechanism, consisting of a slider, moving link, compression spring, torsion spring, and foot. Figure 5C illustrates the gear transmission mechanism, composed of Gear A, Gear B, Gear C, Gear D, rope, winch, and one-way bearings, accompanied by a top view showing the gear arrangement. The working principle of the mechanism is as follows.

To initiate the jumping takeoff mechanism, the drive motor in the gear transmission operates in the forward direction: The one-way bearing at the motor output shaft is in an overrunning state, allowing the motor to drive Gear A, which meshes with Gear B on the intermediate shaft, achieving the first-stage speed reduction with a transmission ratio of 4:1 and amplifying the output torque by a factor of 4. Gear B is rigidly fixed to the intermediate shaft, and Gear C at the lower end of the shaft meshes with Gear D on the output shaft, achieving the second-stage 4:1 speed reduction and further amplifying the torque by another factor of 4. The two-stage reduction stages are arranged in series, resulting in a total transmission ratio of 16:1, and the motor output torque, amplified by a factor of 16, is transmitted to the winch. Under this condition, the one-way bearing inside the winch is in an engaged and locked state, rigidly coupling the output shaft with the winch. The winch rotates synchronously with the shaft to wind the rope; one end of the rope is fixed to the winch, while the other end is connected to the leg slider via a guide shaft, pulling the slider–link mechanism into a flexed configuration, thereby twisting the torsion spring and compressing the compression spring to store elastic potential energy.

Upon completion of energy storage, the drive motor reverses direction: The one-way bearing at the motor end switches to the locked state, keeping the gear transmission system stationary; the one-way bearing at the winch enters the overrunning state, decoupling the winch from the output shaft and instantaneously releasing the rope constraint. The torsion spring and compression spring rapidly rebound to their original positions, releasing the stored elastic potential energy and driving the foot to extend rapidly against the ground, thereby completing the jumping propulsion. The system of springs is therefore considered an engineering equivalent of the elastic energy storage function observed in locust hindlegs rather than a direct reproduction of the biological elastic tissues. Accordingly, the torsion and compression springs used in the robotic mechanism should not be interpreted as direct mechanical replicas of either the locust semi-lunar processes or the extensor tibiae apodemes. In addition, the present mechanism does not incorporate a dedicated unloading function for releasing stored elastic energy without executing the jumping motion; such a safety function will be considered in future prototype development.

### 3.3. Kinematic Analysis of the Bio-Inspired Jumping Takeoff Mechanism

To verify the feasibility of the autonomous jumping takeoff mechanism, a three-dimensional model of the takeoff mechanism was first established in SolidWorks 2025 SP5.0, with model states preserved at each stage. The model was then imported into the Adams (2025) virtual prototype environment for kinematic simulation and analysis at different torsion spring angles (*β*). Adams, as a multibody dynamics simulation platform, has been widely adopted in the kinematic analysis and validation of bio-inspired jumping robots [27,28,29].

**Figure 6:**
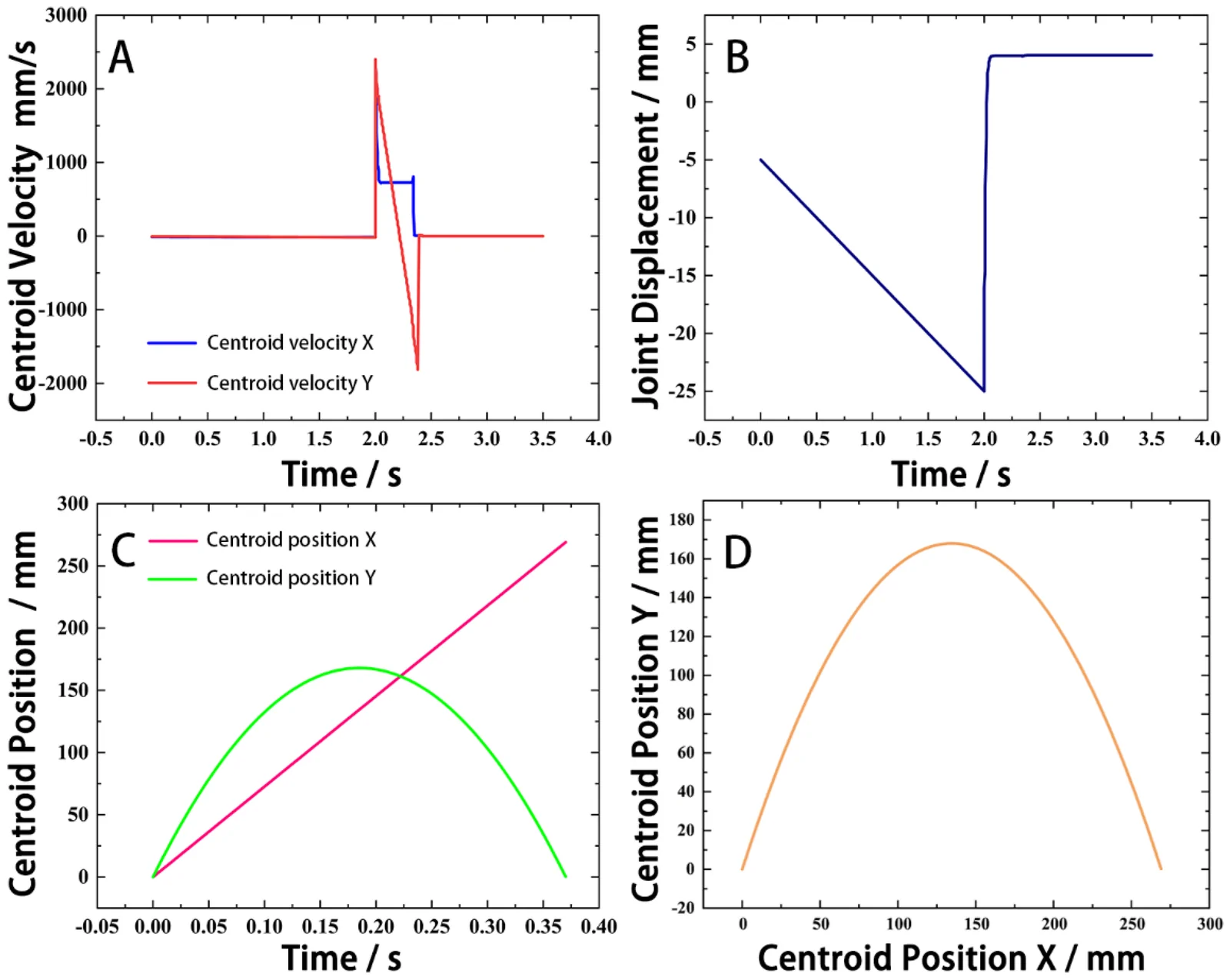
Kinematic simulation analysis of the jumping takeoff mechanism (*β* = 40°). (**A**) CoM velocity; (**B**) The displacement variation at the joint throughout the process; (**C**) Centroid position X and Centroid position Y; (**D**) The trajectory of the centroid.

As shown in Figure 6A, the jump occurs at the second second, with rapid changes in both X- and Y-direction velocities. The entire locking–jumping process is completed within 50 ms, which is highly consistent with the aforementioned biological analysis results. During the jumping process, the maximum velocity in the X-direction reaches 0.727 m/s, and in the Y-direction reaches 1.816 m/s, with a maximum resultant velocity of 1.955 m/s. The duration from takeoff to landing is approximately 0.37 s, which agrees well with the locust’s maximum jumping velocity and jumping process. The locust-inspired mechanism satisfies the takeoff requirements in terms of both response time and maximum resultant velocity. In the kinematic simulation, the jumping process was analyzed under idealized conditions. Ground contact force, foot–ground friction, and aerodynamic drag were not included.

Figure 6B shows the displacement variation at the joint throughout the process. As the motor starts, the rope gradually shortens, bringing the femur closer to the tibia, and the joint moves downward, reaching a maximum displacement of approximately 25.47 mm during the locking phase. When the jumping process begins, it rapidly returns to the initial position. Due to the increase in joint angle, the joint displacement slightly exceeds the initial position by approximately 4.83 mm, which is consistent with biological experiments on locust jumping.

Figure 6C,D show the displacement of the center of mass and its position during the 0.37 s airborne phase. As shown in Figure 6D, the maximum jumping height reaches 168.2 mm, and the horizontal jumping distance reaches 134.6 mm, approximately three times the length of the flapping-wing robot, which is consistent with biological knowledge. During the airborne phase, the center of mass moves at nearly constant velocity in the X-direction, while decelerating uniformly in the Y-direction due to gravity, resulting in a parabolic trajectory.

From the above analysis, it can be concluded that under the idealized simulation conditions, the proposed mechanism at a torsion spring angle of 40° achieves a jumping timescale and resultant velocity comparable to the experimentally observed locust jumping performance. This comparison is intended to evaluate the functional performance of the engineering mechanism rather than to demonstrate a direct reproduction of the complete biological jumping process. The results indicate that β = 40° is sufficient to satisfy the takeoff requirements adopted in the present robotic design.

To provide reliable velocity references for the subsequent Ansys Fluent (2024 R2) aerodynamic simulations, the simulation was performed at increments of 5° for the torsion spring angle. The 5° interval was selected as the parametric resolution for the numerical sensitivity analysis rather than being adopted from a specific previous study. This interval provides sufficient resolution to capture the variation trend of the mechanism performance with torsion spring angle while maintaining a reasonable number of simulation cases and computational cost. At *β* = 40°, the maximum resultant velocity is 1.955 m/s; at *β* = 45°, the maximum resultant velocity is 2.043 m/s; at *β* = 50°, the maximum resultant velocity is 2.270 m/s; at *β* = 55°, the maximum resultant velocity is 2.498 m/s; at *β* = 60°, the maximum resultant velocity is 2.724 m/s; at *β* = 65°, the maximum resultant velocity is 2.951 m/s; at *β* = 70°, the maximum resultant velocity is 3.178 m/s.

## 4. Aerodynamic Performance Analysis of the Bio-Inspired Jumping Takeoff Mechanism

### 4.1. Aerodynamic Performance with Different Angles of Attack

To evaluate the aerodynamic performance of the proposed locust-inspired jumping mechanism during takeoff and to determine the optimal attitude angle for the flapping-wing robot during the takeoff phase, this study employed Ansys Fluent (2024 R2) to conduct numerical simulations of the flow field around the robot fuselage at different angles of attack (hereinafter denoted as *α*). The simulations were based on a pressure-based solver with the Shear Stress Transport k–omega (SST k−ω) turbulence model. The inflow velocity was set to 3.18 m/s (corresponding to the maximum initial velocity in the Adams simulation), and a no-slip boundary condition was applied to the fuselage surface. The effects of the angle of attack on aerodynamic characteristics were quantitatively analyzed by monitoring lift, drag, lift-to-drag ratio, as well as velocity distribution, turbulence kinetic energy (TKE), and eddy viscosity in the flow field [30,31,32]. A three-dimensional computational domain with dimensions of 320 mm × 350 mm × 280 mm was established around the robot model. The computational domain was designed to minimize the influence of external boundary effects on the aerodynamic results. Considering the complex geometry of the bio-inspired jumping mechanism, an unstructured tetrahedral mesh was adopted for flow field discretization. Local mesh refinement was applied to the leg structures, where significant flow variations and complex fluid interactions occur during the takeoff process. The maximum mesh size on the leg surface was refined to 0.0005 m, while the minimum mesh size within the computational domain was set to 0.005 m. Three prism layers were generated near the robot surface to improve the resolution of near-wall flow characteristics. The final computational mesh consisted of approximately 3.2 million cells. The SST k–ω turbulence model was selected because of its capability to accurately predict flow separation and adverse pressure gradient effects around complex aerodynamic structures. To ensure the reliability and independence of the numerical results, a mesh independence analysis was conducted using different mesh resolutions. The aerodynamic parameters obtained from the medium and fine meshes showed negligible differences, with variations of less than 1%, indicating that the selected mesh density was sufficient for subsequent simulations. Furthermore, the near-wall resolution was evaluated using the dimensionless wall distance (y+) value (below 5), which remained within the recommended range for the SST k–ω turbulence model, ensuring reliable prediction of boundary layer characteristics. The lift and drag coefficients were computed using the standard dynamic-pressure formulation:

$$C_{L}=\frac{F_{L}}{\frac{1}{2}\rho V_{\infty }^{2}A_{ref}}$$

$$C_{D}=\frac{F_{D}}{\frac{1}{2}\rho V_{\infty }^{2}A_{ref}}$$

where $F_{L}$ and $F_{D}$ are the lift and drag forces obtained by integrating the pressure and shear stress distributions over the robot surface in ANSYS Fluent (2024 R2); ρ=1.225 kg/m^3^ is the air density at standard atmospheric conditions; $V_{\infty }=3.18$ m/s is the freestream velocity, corresponding to the maximum takeoff velocity obtained from the Adams dynamic simulation; and $A_{ref}$ is the reference area, defined as the maximum projected area of the robot fuselage in the direction perpendicular to the freestream. The lift force $F_{L}$ is defined as the force component perpendicular to the freestream direction, while the drag force $F_{D}$ is the force component parallel to the freestream direction. These coefficients were computed automatically by ANSYS Fluent (2024 R2) based on the pressure and viscous forces acting on the selected wall surfaces.

**Figure 7:**
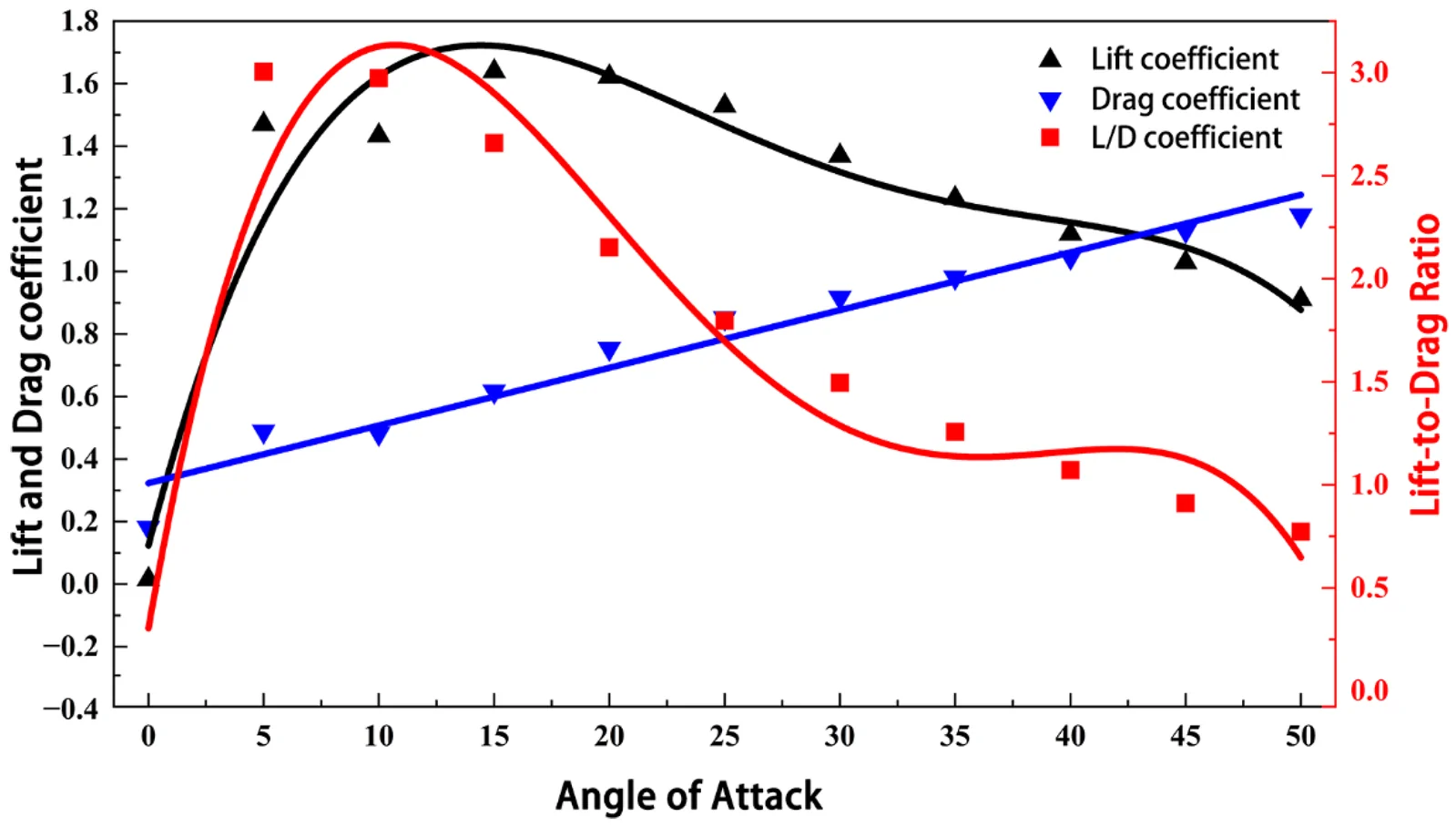
Aerodynamic performance analysis of the bio-inspired jumping mechanism at different *α*s. As the angle of attack increases, the drag coefficient continuously increases, while the lift coefficient and lift-to-drag ratio initially increase and then decrease, reaching their peak values at *α* = 15° and *α* = 5°, respectively.

As shown in Figure 7, the angle of attack has a significant influence on the lift-to-drag ratio. At *α* = 0°, the lift-to-drag ratio reaches its minimum value of 0.877, indicating that the wing surface fails to generate effective lift under this condition. At *α* = 5°, the lift-to-drag ratio reaches a maximum of 3.005, with a lift coefficient of 1.470 and a drag coefficient of only 0.489, demonstrating that the lift generated per unit drag is highest at this angle, corresponding to optimal aerodynamic efficiency. As the angle of attack continues to increase, the lift coefficient continues to rise and reaches a peak of 1.640 at approximately *α* = 15°, but the drag coefficient increases more significantly, causing the lift-to-drag ratio to decrease progressively: it drops to 2.974 at *α* = 10°, further declines to 1.495 at *α* = 30°, and diminishes to only 0.911 at *α* = 50°.

To further reveal the flow field evolution at different *α*, the eddy viscosity contours, velocity contours, and TKE contours were extracted.

**Figure 8:**
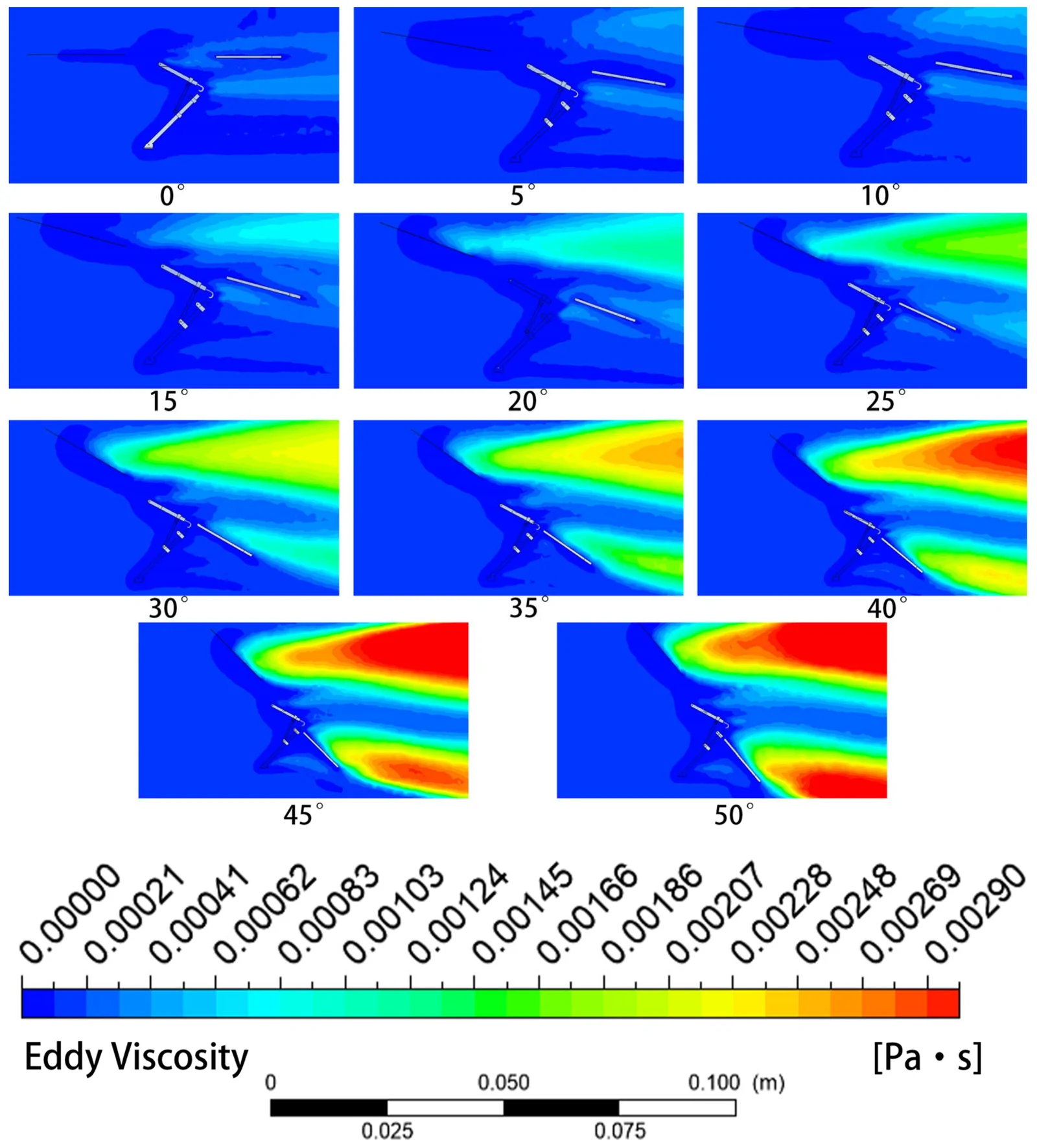
Eddy viscosity contours at different *α*s. As *α* increases from 0° to 50°, the eddy viscosity transitions from extremely low levels (dark blue) in attached flow to high levels (red/orange) in fully separated flow. At 5°, the flow remains predominantly laminar with minimal turbulence; at 15–20°, separation bubbles with elevated eddy viscosity appear; at 30° and above, massive turbulent recirculation zones form, indicating deep stall.

As shown in Figure 8, the eddy viscosity contours reflect the evolution from laminar attached flow to fully turbulent separated flow as *α* increases from 0° to 50°. At 0°, the overall flow field appears dark blue, indicating extremely low eddy viscosity, with only a faint light blue trace along the narrow centerline of the wake, suggesting almost no turbulence generation. At 5°, the field remains predominantly dark blue, with only extremely weak light blue spots appearing above the trailing edge, indicating slight boundary layer instability while the overall flow remains at a low turbulence level. At 10°, the light blue region in the wake expands slightly, particularly at the trailing edge of the upper surface, where the shear layer strength begins to increase. At 15°, the changes become more pronounced, with a cyan/light green region appearing behind the upper surface, indicating that flow separation leads to a significant rise in eddy viscosity within the separation bubble. At 20°, the high eddy viscosity region (green) expands further and extends downstream, with a broadened wake and large-scale separation inducing intense turbulent dissipation. At 25°, a yellow/orange core appears in the separated shear layer, indicating the onset of stall and generating high-intensity turbulent fluctuations. At 30°, the high eddy viscosity region occupies a large space above the model, with large-scale separated vortices and intense internal mixing, exhibiting characteristics of deep stall. At 35°, an orange-red region appears and remains relatively fixed near the upstream separation point, while the downstream wake also shows elevated eddy viscosity, indicating that the flows on both the upper and lower surfaces are severely disturbed. At 40°, the entire upper half of the robot is covered by high eddy viscosity, with the mainstream completely detached and forming a massive turbulent recirculation zone. At 45°, the red region reaches its peak, and orange patches also appear in the downstream wake, indicating extremely chaotic flow with substantial energy dissipation. At 50°, the extensive red and orange regions persist at very high levels, with pressure drag dominating and turbulent mixing reaching its limit state.

**Figure 9:**
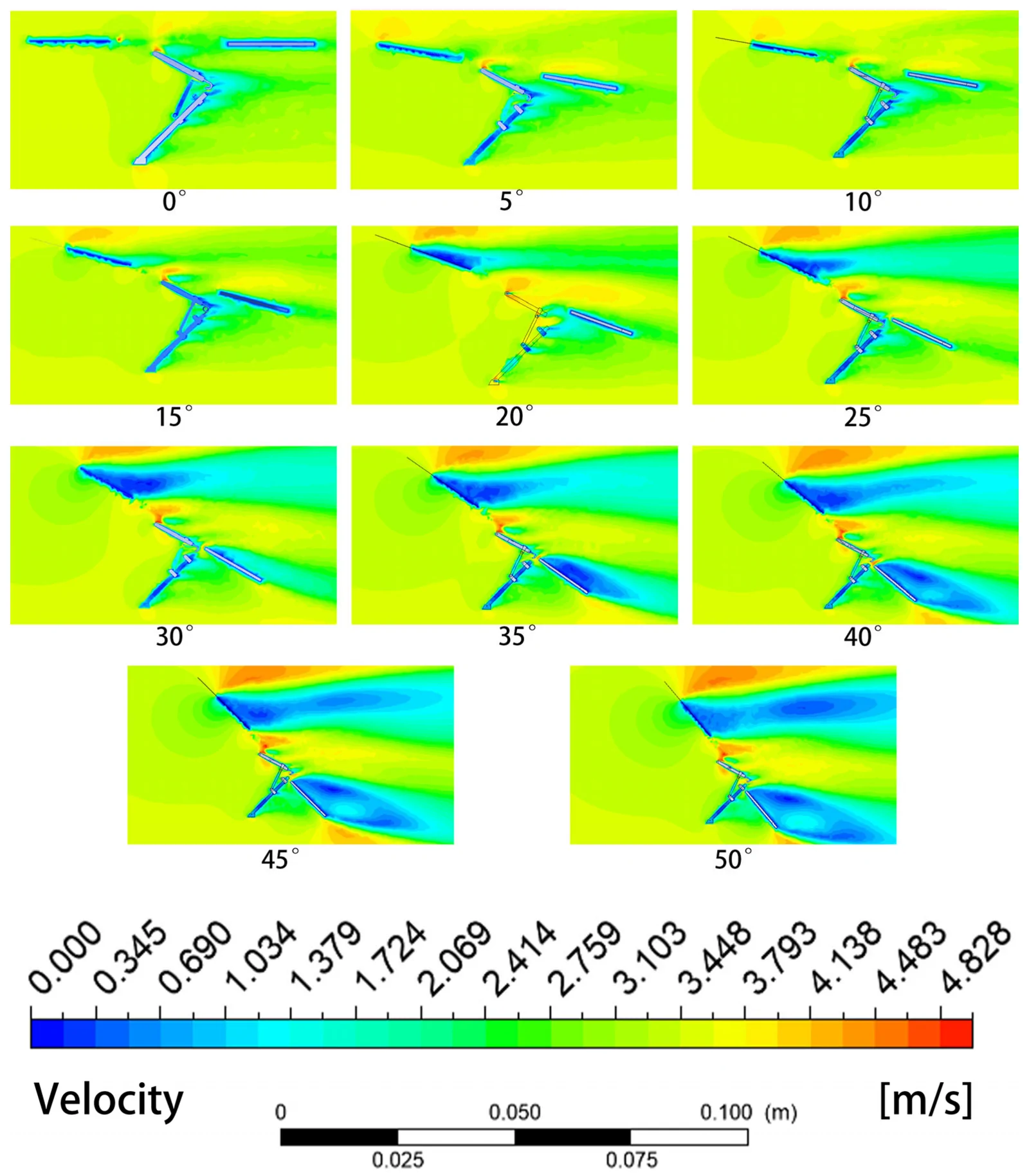
Velocity contours at different *α*s. As *α* increases from 0° to 50°, the velocity field evolves from well-attached flow to fully separated flow. At 5°, a small wedge-shaped low-velocity region appears at the trailing edge as an initial separation sign; at 20°, a large separation bubble occupies nearly half of the upper surface; at 40–50°, the mainstream completely detaches, forming extensive low-velocity wakes and the aerodynamic performance becomes dominated by form drag.

As shown in Figure 9, the velocity contours present a complete evolution from attached flow to fully separated flow as *α* increases from 0° to 50° [33,34,35]. At *α* = 0°, the background appears uniformly light green, with only a thin dark blue boundary layer around the model and rapid recovery in the wake, indicating well-attached flow. When *α* increases to 5°, the flow velocity on the upper surface increases slightly, while the velocity on the lower surface decreases, and a small dark blue wedge-shaped region appears in the wake, indicating the initial signs of flow separation. At 10°, the high-velocity region on the upper surface becomes more pronounced, and the dark blue separation region in the wake expands and extends downstream, forming a relatively small dead-water zone. At 15°, separation intensifies, with a large dark blue low-velocity region appearing on the rear portion of the upper surface, and the mainstream is pushed away from the surface, with the high-velocity yellow-green region concentrated outside the separated shear layer. At 20°, the dark blue separation bubble expands significantly, occupying nearly half of the space above the model, exhibiting typical pre-stall characteristics. At 25°, a massive dark blue region envelops the upper surface, with only a yellow-green band representing high-velocity flow visible at the edge of the outermost shear layer, indicating fully developed flow separation. At 30°, the separation region becomes extremely wide, and the upstream high-velocity flow sweeps directly over the top of the model without any contact with the surface. At 35°, the separation region becomes more pronounced, with additional blue areas appearing in the lower surface flow due to disturbances. At 40°, almost the entire upper half of the model appears dark blue, indicating extremely low-velocity regions, with the freestream velocity color preserved only in the far field. At 45°, the low-velocity region not only covers the upper surface but also forms a very wide dark blue wake directly behind the model, indicating substantial loss of fluid kinetic energy. At 50°, the extreme separation causes the model to be surrounded by a massive low-velocity region, with the velocity field symmetry completely destroyed and the aerodynamic performance dominated primarily by form drag.

**Figure 10:**
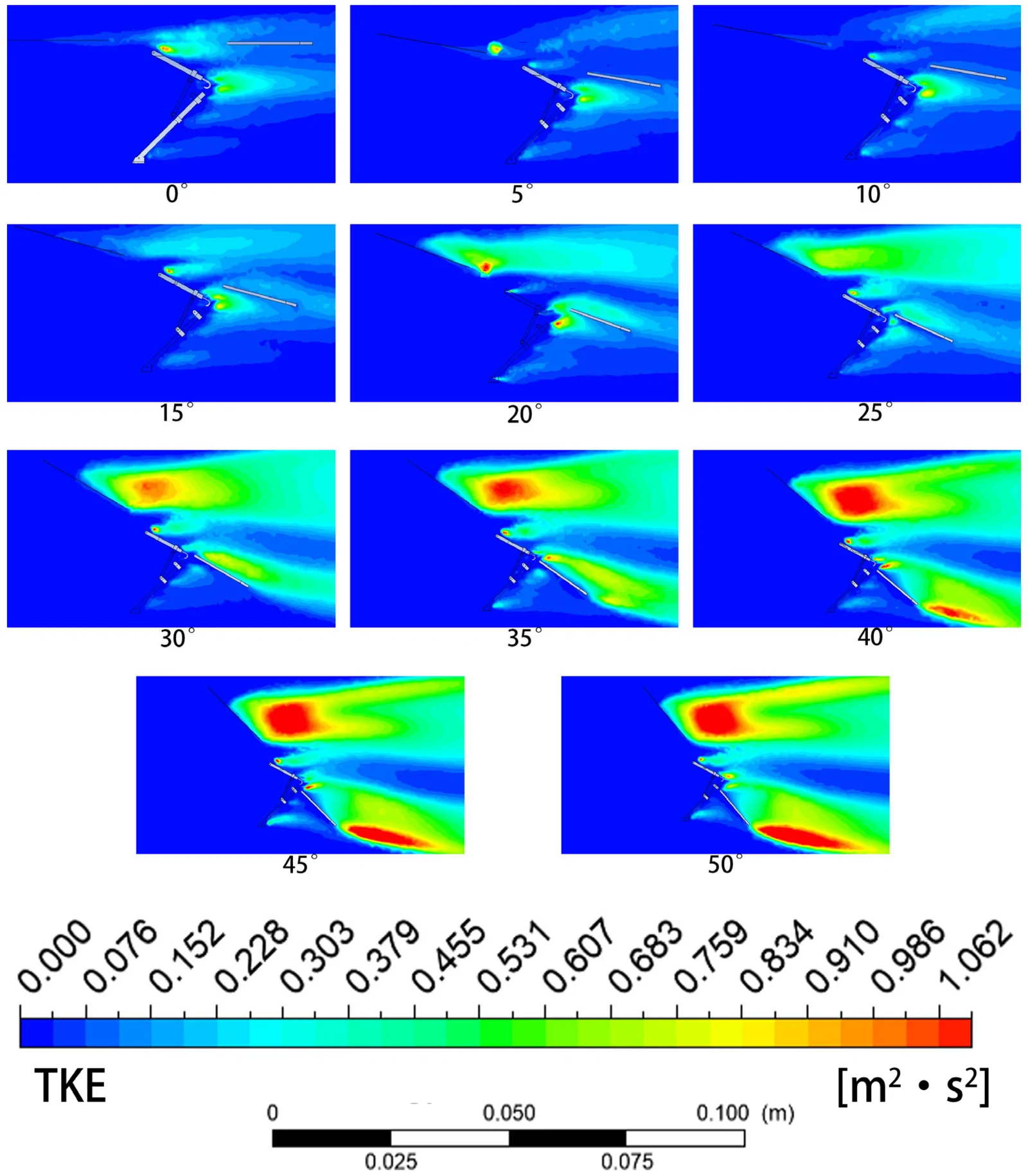
TKE contours at different *α*s. As *α* increases from 0° to 50°, the TKE evolves from near-zero levels to extremely high energy densities. At 5°, small TKE spots appear at the trailing edge, indicating boundary layer transition; at 15–20°, a distinct yellow core appears at the separated shear layer; at 25° and above, the core transitions to orange/red, acting as a high-energy turbulent source; at 50°, the entire upper flow field is filled with intense red TKE regions, indicating maximum energy dissipation.

As shown in Figure 10, the TKE contours present the evolution from laminar flow to highly turbulent flow as *α* increases from 0° to 50°. At 0°, the overall field appears dark blue, with the TKE close to zero, and only faint cyan spots visible at the model tip and along the extremely thin wake line, indicating very clean flow. At 5°, two distinct cyan/light green spots appear at the trailing edge of the upper surface and the tip of the wake, corresponding to boundary layer transition or small vortices generated by incipient separation. At 10°, the high-TKE spots on the upper surface become larger and brighter, with enhanced instability within the separation bubble and increasingly active turbulent fluctuations. At 15°, a distinct yellow core appears at the initial section of the separated shear layer, where the strong velocity shear between the separated flow and the quiescent air excites high-energy turbulence. At 20°, the yellow core becomes brighter and is surrounded by large green regions, with the high-TKE zone concentrated near the separation point where the vortex shedding frequency is highest. At 25°, the core region transitions to orange/red, with extremely high energy density, acting like an engine that continuously delivers turbulent energy downstream, resulting in highly chaotic wake. At 30°, the red high-TKE core is highly concentrated and intense, located slightly above the model, indicating that the separated shear layer still maintains a high energy level. At 35°, the red region persists, and orange high-energy zones also appear in the downstream wake, indicating that both upper and lower surface flows begin to become unstable. At 40°, massive red patches dominate the upper flow field, with extremely intense turbulent fluctuations serving as the primary source of aerodynamic noise and vibration. At 45°, the red regions merge into a continuous broad area, and orange TKE bands also appear at the edges of the downstream wake, indicating that the entire flow field is in a highly turbulent state. At 50°, the extremely high-intensity red TKE regions persist, with the flow field filled with large-scale coherent structures and small-scale random fluctuations, resulting in extremely high energy dissipation rates.

Within the range of 0° to 50°, *α* = 5° demonstrates the optimal aerodynamic performance [34]. At this angle of attack, the lift-to-drag ratio reaches its maximum value of 3.005, the flow remains attached, and the airflow conforms well to the surface, with only extremely weak boundary layer instability appearing at the trailing edge. Both the eddy viscosity and TKE remain at low levels, indicating stable flow with minimal energy dissipation. When *α* exceeds 15°, flow separation on the upper surface intensifies, the separation bubble expands significantly, and the eddy viscosity and TKE rise sharply, causing a rapid decline in the lift-to-drag ratio and a substantial reduction in aerodynamic efficiency. Therefore, *α* = 5° is determined as the optimal angle of attack for the flapping-wing robot during the jumping takeoff phase.

### 4.2. Aerodynamic Performance with Different Torsion Spring Angles

**Figure 11:**
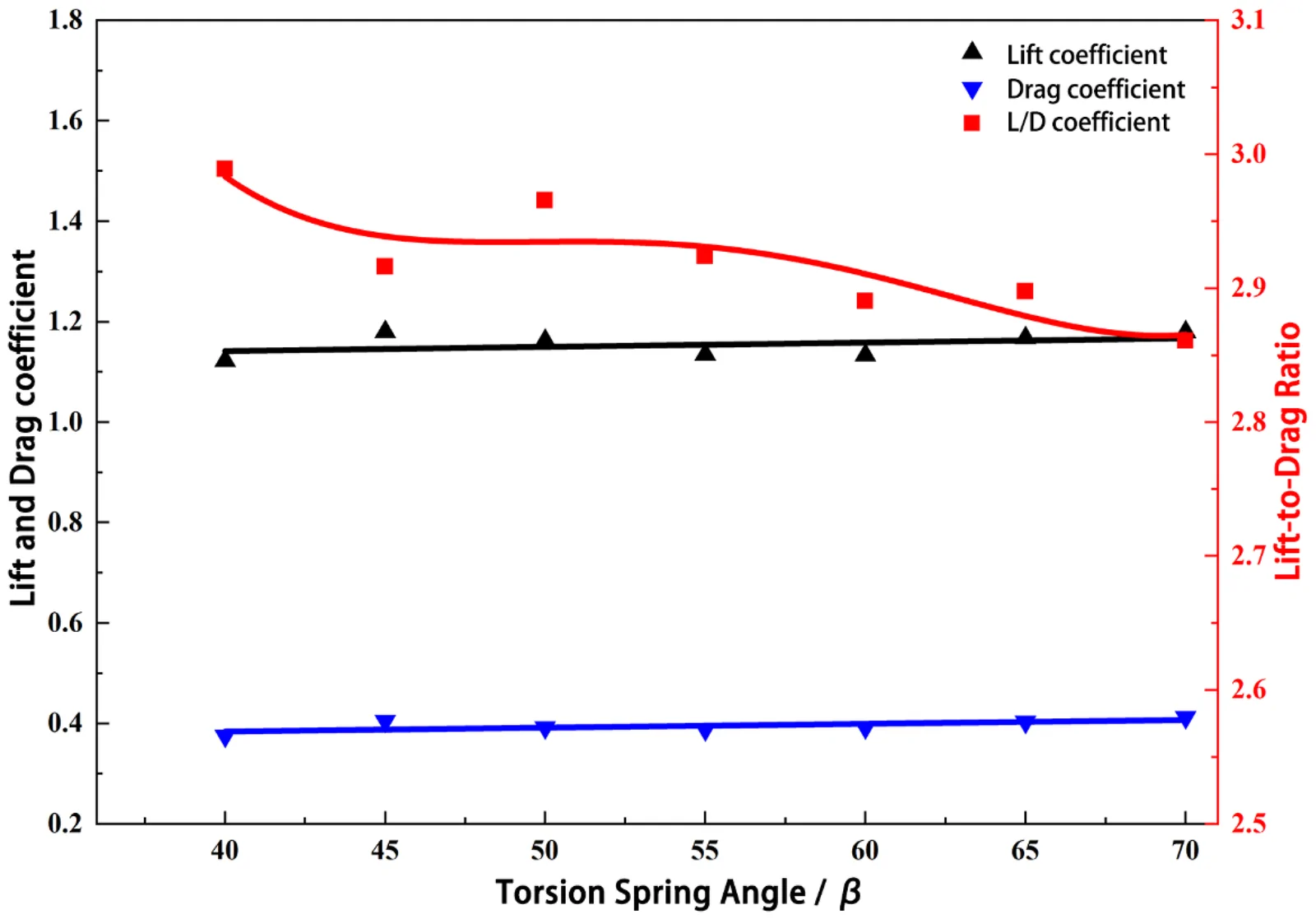
Aerodynamic performance analysis of the bio-inspired jumping mechanism at *α* = 5° for varying *β*s. As *β* increases from 40° to 70°, the drag coefficient continuously increases; the lift coefficient shows little variation; the lift-to-drag ratio reaches its peak at *β* = 40° and gradually declines thereafter. This indicates that *β* = 40° provides the optimal lift-to-drag ratio.

Based on the optimal angle of attack (*α* = 5°) obtained from the above analysis, the effect of *β* on the flow field during jumping takeoff was further investigated. Through simulation analysis, the eddy viscosity contours, velocity contours, and TKE contours were extracted. As shown in Figure 11, the lift-to-drag ratio reaches its peak at β = 40° and gradually declines as β increases from 40° to 70°.

**Figure 12:**
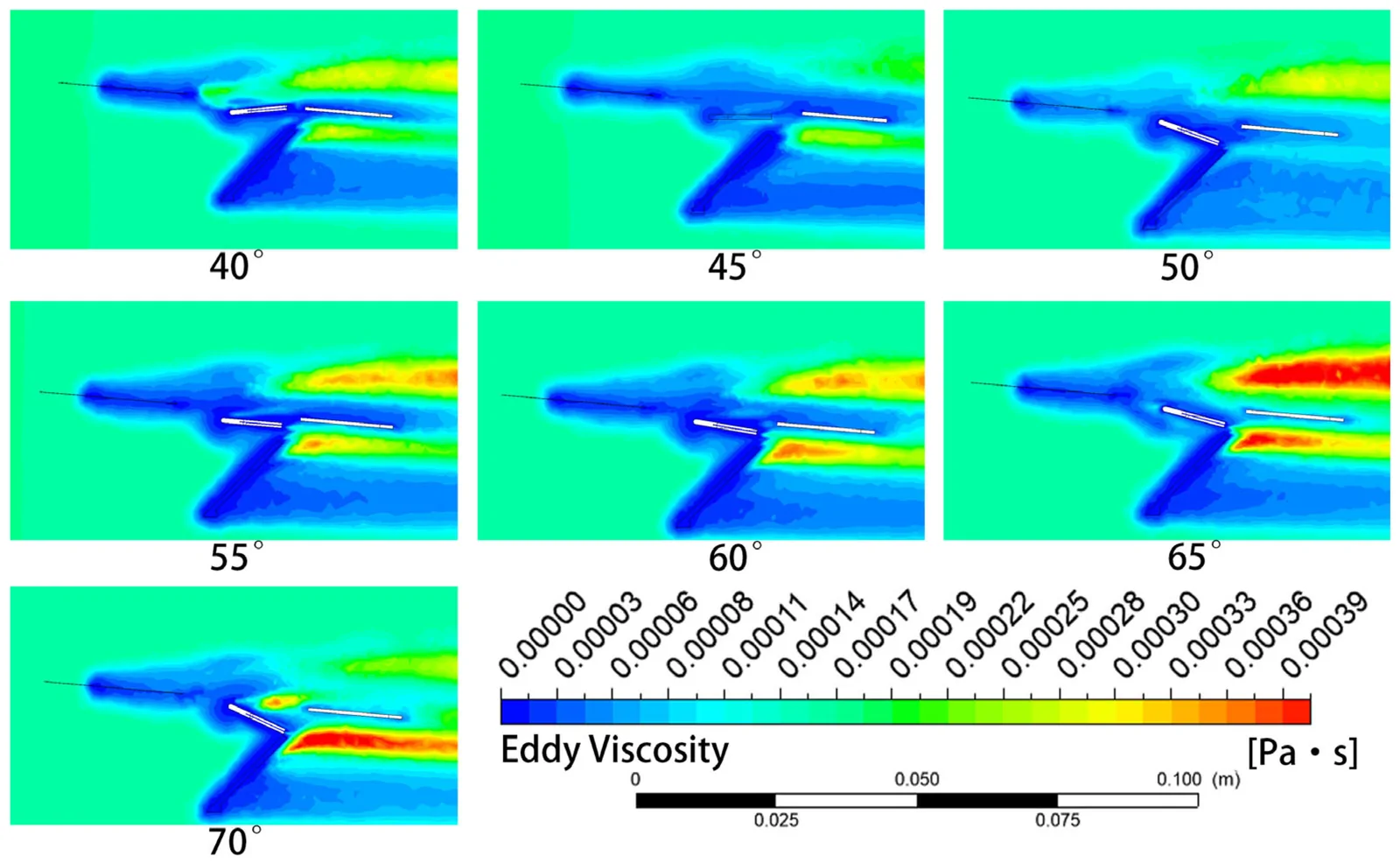
Eddy viscosity contours at *α* = 5° for varying *β*s. As *β* increases from 40° to 70°, the flow separation and turbulence within the joint angle and on the leeward side of the tibia progressively intensify. At 40°, a small orange-yellow high-eddy-viscosity region appears at the joint angle; at 50°, turbulent streaks emerge on the tibia’s leeward side; at 60°, the wake expands into dark green/yellow regions; at 70°, the wake becomes extremely broad with large red/orange patches, indicating the formation of a von Kármán vortex street and maximum turbulent mixing.

As shown in Figure 12, the eddy viscosity contours clearly illustrate the progressive intensification of flow separation and turbulence within the joint angle and on the leeward side of the tibia as *β* increases from 40° to 70°. At *β* = 40°, a distinct orange-yellow high-eddy-viscosity region appears at the angle formed between the posterior side of the femur and the anterior side of the tibia. At this stage, the joint bending is relatively small, and the airflow undergoes significant stagnation and recirculation within this “V”-shaped region, resulting in strong shear and mixing, while the boundary layer on the leading edge of the femur remains thin and blue. At *β* = 45°, the yellow high-eddy-viscosity region within the joint angle persists but contracts slightly in extent, and the wake above the femur begins to show weak light green disturbances, indicating that the flow compression effect remains significant but is increasingly influenced by downstream flow. At *β* = 50°, a turning point is reached: the high-eddy-viscosity region within the joint angle becomes more concentrated and brighter, while light green turbulent streaks begin to appear on the leeward side of the tibia, marking the onset of flow separation on the tibia’s leeward surface. At *β* = 55°, the orange region within the joint angle remains prominent, and the wake behind the tibia widens considerably, with large green/yellow regions appearing, indicating that as the leg bends further, the tibia increasingly obstructs the airflow, generating a broader low-pressure separation zone downstream with intensified turbulence. At *β* = 60°, the orange-red high-eddy-viscosity region within the joint angle becomes very intense, and the wake behind the tibia expands further, shifting to dark green and locally yellow. The large joint angle turns both the femur and tibia into strong sources of flow disturbance, with the separation bubble behind the tibia having developed substantially. At *β* = 65°, the overall turbulence level of the entire flow field rises, with the red core at the joint angle becoming very bright, and the wake behind the tibia occupying most of the lower-right region, appearing yellow-green. This represents a high-drag state, where the airflow around the complex limb structure generates significant energy dissipation. At *β* = 70°, the maximum angle in this group is reached: not only does a high-eddy-viscosity region exist within the joint angle, but the wake behind the tibia becomes extremely broad, displaying large red/orange patches. The extreme bending causes the airflow to completely detach from the posterior surface of the tibia, forming a massive von Kármán vortex street-like shedding zone, with turbulent mixing reaching its peak and aerodynamic drag at its maximum [36].

**Figure 13:**
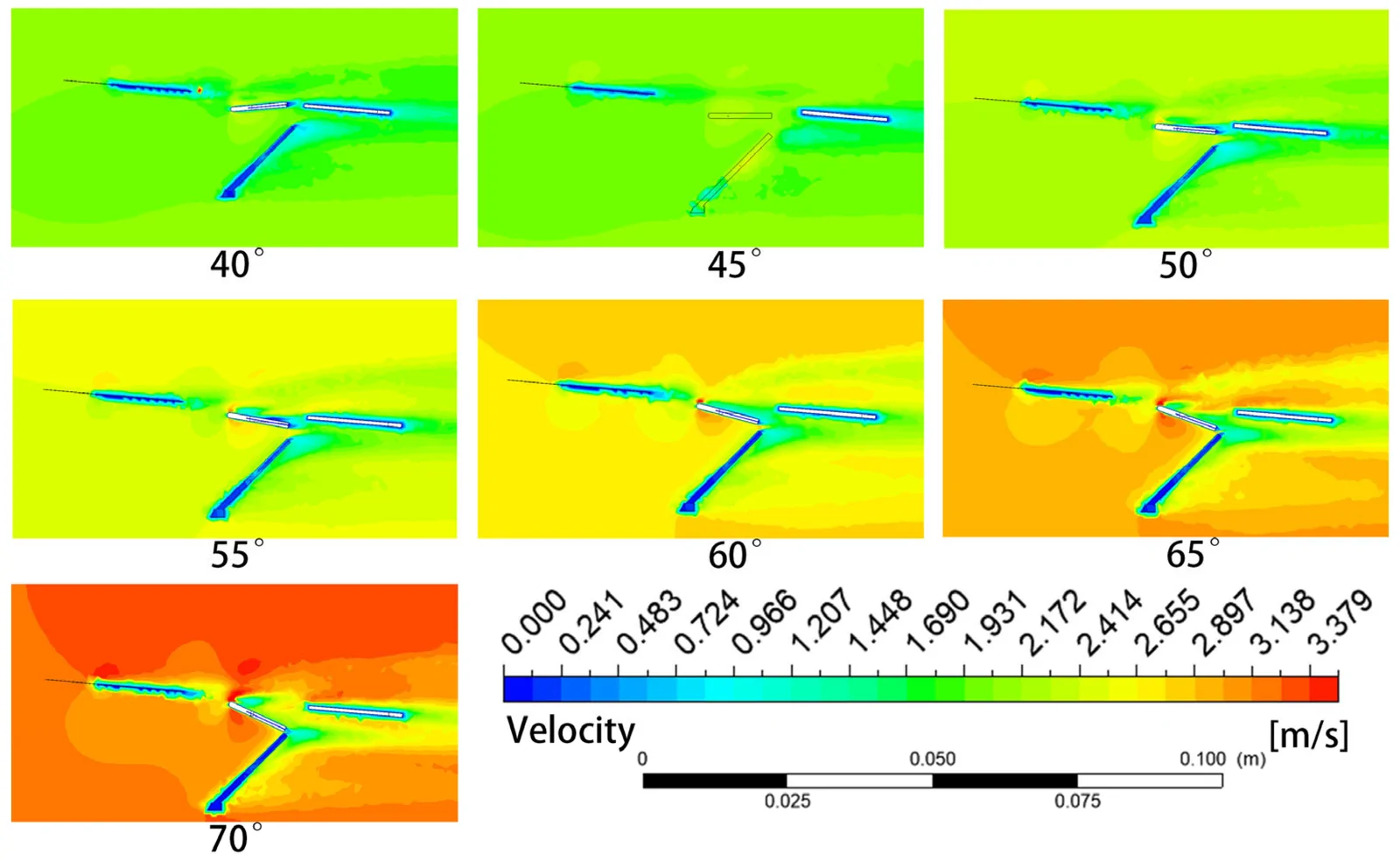
Velocity contours at *α* = 5° for varying *β*s. As *β* increases from 40° to 70°, the velocity field evolves from localized low-velocity zones to typical bluff-body flow, and further to highly asymmetric high-speed jets with extensive wakes. At 40°, the flow primarily passes over the femur and beneath the tibia, with a stagnant dead-water zone in the joint angle; at 60°, a high-velocity orange zone appears above the femur with a large low-velocity wake behind; at 70°, an extremely fast orange-red jet forms above the femur while the rear regions are occupied by extensive dark blue low-velocity zones, indicating strong shear forces and high pressure drag.

As shown in Figure 13, the velocity contours clearly illustrate the evolution from localized low-velocity zones to typical bluff-body flow, and further to highly asymmetric high-speed jets with extensive wakes, as *β* increases from 40° to 70°. At *β* = 40°, the incoming flow velocity is relatively high (green/yellow), with the flow accelerating to bright yellow at the leading edge of the femur, while forming a dark blue low-velocity zone deep within the angle between the femur and tibia. The wake behind the tibia remains narrow, with the flow primarily passing over the femur and beneath the tibia, while the interior of the joint angle acts as a relatively stagnant dead-water zone. At *β* = 45°, the overall velocity distribution is similar to that at 40°, with the yellow acceleration zone on the upper surface of the femur still prominent and the blue low-velocity zone within the joint angle persisting, indicating a stable flow structure. At *β* = 50°, a larger dark blue region appears behind the tibia compared to previous angles, while the high-velocity zone (yellow) at the leading edge of the femur remains strong, indicating that as the angle increases, the tibia increasingly obstructs the airflow, forming a larger low-pressure, low-velocity separation zone on its leeward side. At *β* = 55°, the blue region within the joint angle becomes deeper and larger, and the blue wake behind the tibia continues to expand and extend far downstream, with the “wind-catching” effect of the limb configuration enhancing the capture of low-velocity fluid. At *β* = 60°, the velocity gradient around the entire model increases significantly: the region above the femur becomes a high-velocity orange zone, while a large blue-cyan low-velocity zone appears behind the model (to the right), exhibiting typical bluff-body flow characteristics—deceleration at the front, acceleration over the top, and a massive wake formation downstream. At *β* = 65°, the dark blue stagnation point at the leading edge of the femur becomes very pronounced, with the flow forced to divert upward and downward. The blue region behind the tibia becomes extremely broad, and the increased effective frontal area leads to greater conversion of kinetic energy into pressure energy at the leading edge, with corresponding losses in the downstream wake. At *β* = 70°, the velocity field exhibits strong asymmetry, with an extremely fast orange-red jet forming above the femur, while the interior and rear regions enclosed by the limb are occupied by extensive dark blue low-velocity zones. This substantial velocity difference indicates strong shear forces and consequently extremely high pressure drag.

**Figure 14:**
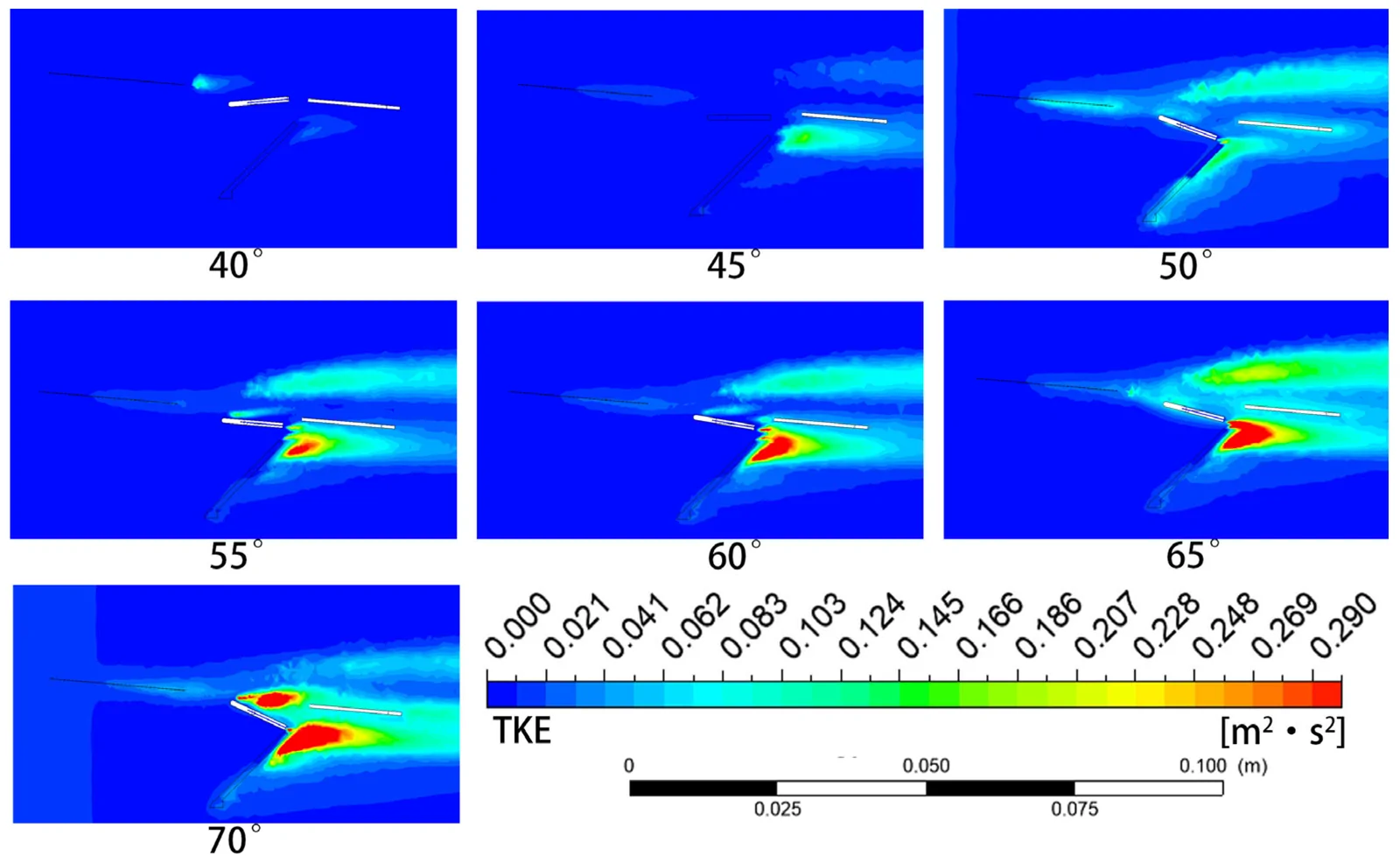
TKE contours at *α* = 5° for varying *β*s. As *β* increases from 40° to 70°, the TKE evolves from low-level fluctuations to a fully turbulent wake. At 40°, the flow remains relatively steady with minimal TKE; at 50°, an orange-red TKE core appears within the joint angle, indicating vortex breakdown; at 60°, two primary high-TKE regions emerge (joint angle core and wake behind tibia); at 70°, the entire wake field is dominated by deep red/orange patches with the shear layer above the femur turning yellow, representing a fully developed turbulent wake and the lowest aerodynamic efficiency.

As shown in Figure 14, the TKE contours clearly illustrate the evolution from low-level fluctuations to a fully turbulent wake as *β* increases from 40° to 70°. At *β* = 40°, the overall background appears dark blue, with only a faint light blue/cyan spot at the leading edge tip of the femur and a small weak green region within the joint angle. The flow remains relatively steady at this stage; although recirculation exists within the joint angle, the turbulent fluctuations are not particularly intense. At *β* = 45°, the TKE at the leading edge of the femur increases slightly, and the green spot within the joint angle becomes more distinct, indicating that minor angular adjustments cause slight shifts in the separation point position, leading to locally enhanced turbulence. At *β* = 50°, a critical turning point is reached: a distinct orange-red TKE core appears within the joint angle, indicating that the recirculating vortex within the angle has become highly unstable and begins to break down vigorously, generating intense turbulent fluctuations. At *β* = 55°, the red high-TKE region within the joint angle becomes larger and brighter, while yellow-green TKE bands begin to appear in the wake behind the tibia. Flow separation intensifies not only within the joint angle but also breaks out fully on the posterior surface of the tibia. At *β* = 60°, two primary high-TKE regions emerge: the red core within the joint angle and a yellow band in the wake behind the tibia. The limb acts as a complex flow disturber, continuously injecting turbulent energy into the flow field. At *β* = 65°, the red region within the joint angle becomes highly saturated, and the TKE in the wake behind the tibia also reaches high levels. The airflow becomes extremely chaotic, not only generating substantial drag but also potentially causing unstable aerodynamic force fluctuations. At *β* = 70°, the TKE throughout the entire field reaches its maximum: the joint angle interior appears deep red, the region behind the tibia displays extensive red/orange patches, and even the shear layer above the femur turns yellow. This represents a fully developed turbulent wake field, where all smooth flow structures are disrupted, and a large amount of energy is dissipated in turbulent fluctuations, making this the state of lowest aerodynamic efficiency.

At *α* = 5° and *β* = 40°, the recirculation zone within the joint angle is relatively small, the wake is narrow, and both the eddy viscosity and TKE remain at low levels, indicating stable flow. As the torsion spring angle increases to 50° and above, flow separation on the leeward side of the tibia intensifies, the turbulence intensity in the wake region increases significantly, and the aerodynamic drag rises rapidly. Combining the analyses from both perspectives, the combination of *α* = 5° and *β* = 40° provides sufficient lift while achieving the best flow attachment and minimal turbulent dissipation and is therefore identified as the optimal aerodynamic configuration for the flapping-wing robot during the jumping takeoff phase.

To further place the present results in the context of previous biological and bio-inspired jumping studies, representative jumping performance and design characteristics are compared in Table 2. Classical biological studies have reported takeoff velocities ranging from approximately 1.4 to 3.2 m/s for locusts under different species and substrate conditions, illustrating both the high-power output and environmental adaptability of the biological jumping system [3,6,26]. Previous bio-inspired jumping robots have mainly focused on reproducing leg-extension kinematics, improving elastic energy-storage capacity, or enhancing takeoff stability [19,27]. For example, the locust-inspired mechanism developed by Mo et al. achieved a jump height of approximately 0.55 m and a horizontal distance of approximately 0.70 m, with particular emphasis on reducing body rotation after takeoff [19], whereas Bai et al. reported a spring-driven miniature jumping robot with a takeoff velocity of 2.21 m/s and a jump height of 220 mm [27]. In comparison, the present mechanism achieves a resultant velocity of 1.955 m/s within a 50 ms jumping process, which falls within the characteristic velocity range reported for biological locust jumping. It should be emphasized that the objective of the present design is not to maximize ballistic jumping height or distance, but to provide a compact and controllable auxiliary takeoff mechanism for a FWARs. More importantly, unlike the representative jumping robots listed in Table 2, the present study further couples the mechanical jumping configuration with aerodynamic analysis and identifies the combination of *α* = 5° and *β* = 40° as providing the highest lift-to-drag ratio. This integrated consideration of biological kinematics, controllable energy storage and release, and aerodynamic performance constitutes the principal engineering distinction of the proposed system. Most previous jumping studies focused on mechanical jumping performance, while aerodynamic evaluation during the ground-to-air transition remains rarely investigated.

Limitations and Future Work: This study has revealed the kinematic characteristics of locust jumping takeoff on slopes of different angles through biological experiments, and based on these findings, proposed a bio-inspired jumping takeoff mechanism with validated dynamic and aerodynamic performance through simulations. However, due to the focused scope of this research and current limitations, the following aspects require further investigation. First, the biological experiments in this study were conducted exclusively on adult male *Locusta migratoria* specimens; the effects of ecological phase differences and individual variations on jumping performance have not been included in the analysis, which may limit the generalizability of the kinematic conclusions. Second, both the dynamic and aerodynamic simulations were performed under idealized boundary conditions and did not fully account for real-world factors such as nonlinear ground friction, joint clearances, and air compressibility effects, which may introduce discrepancies between the simulation results and the actual performance of physical prototypes. In future work, a physical prototype will be fabricated based on the optimal aerodynamic configuration (*α* = 5°, *β* = 40°) identified in this study, and ground jumping and takeoff transition experiments will be conducted to validate the simulation results. Furthermore, a flapping-wing aerodynamic force and attitude control module will be integrated to investigate the timing and coordination strategies for wing engagement after liftoff, ultimately achieving fully autonomous multimodal transition from static standing to stable flight. In addition, adaptive takeoff strategies for different landing surfaces (e.g., hard ground, vegetation layers, and soft soil) and active jump height regulation mechanisms will also be key directions for subsequent research. In addition, the current robotic mechanism does not reproduce the biological unloading function that allows the loaded elastic system to be relaxed without completing a jump. A controllable mechanical unloading or energy-dissipation mechanism will therefore be considered in future prototype development. The biological kinematic results also exhibited inter-individual and inter-trial variability; consequently, the present biological experiments were used primarily to identify functional trends and performance ranges rather than exact deterministic design parameters.

## 5. Conclusions

In this study, the Asian migratory locust (*Locusta migratoria*) was selected as the biological prototype to investigate the functional principles underlying insect jumping takeoff. High-speed camera experiments were conducted to capture the jumping process of locusts on slopes ranging from 0° to 40°, and the corresponding kinematic characteristics were systematically analyzed. The results show that as the slope angle increased from 0° to 30°, the tarsal displacement increased from 7.1 mm to 8.6 mm, the tibial vertical velocity increased from 1.25 m/s to 1.85 m/s, and the horizontal velocity increased from 0.85 m/s to 1.65 m/s. Meanwhile, the takeoff angle decreased from approximately 90° to about 50°. At 30°, the velocity curves of the femur and tibia exhibited strong synchronization, indicating improved hindleg coordination. When the slope angle reached 40°, the tarsal displacement decreased to 7.9 mm, accompanied by a brief backward sliding phenomenon before takeoff, suggesting reduced jumping stability under the steepest tested condition.

Based on the observed kinematic characteristics and the functional energy storage–release strategy of locust hindlegs, a bio-inspired jumping takeoff mechanism was developed for a flapping-wing robot. The proposed mechanism does not attempt to fully replicate the physiological structures of locust jumping but rather extracts the key mechanical principles of elastic energy accumulation, mechanical locking, and rapid energy release. The mechanism employs a gear transmission system with a total reduction ratio of 16:1 to amplify torque and uses forward and reverse motor rotation to switch two sets of one-way bearings between locking and overrunning states, enabling controllable energy storage and rapid release. Adams dynamic simulations demonstrate that at a torsion spring angle of β = 40°, the mechanism completes the jumping process within 50 ms, achieving a maximum resultant velocity of 1.955 m/s, a jump height of 168.2 mm, and a horizontal displacement of 134.6 mm.

The aerodynamic characteristics of the flapping-wing robot during the jumping takeoff phase were further investigated using Ansys Fluent (2024 R2) under different angle-of-attack and torsion spring conditions. The results indicate that an angle of attack of α = 5° provides the highest lift-to-drag ratio of 3.005, accompanied by attached flow and relatively low TKEand eddy viscosity. When the angle of attack exceeded 15°, flow separation became more significant, resulting in a rapid decrease in aerodynamic efficiency. The torsion spring angle analysis further revealed that β = 40° provides a stable flow field with a smaller recirculation region and narrower wake, whereas larger torsion spring angles induce intensified flow separation and increased aerodynamic drag. Therefore, the combination of α = 5° and β = 40° was identified as the optimal configuration for the jumping takeoff phase. 

Overall, this study presents a functional bio-inspired jumping takeoff mechanism inspired by the efficient energy-management strategy of locust hindlegs. From an engineering perspective, the combination of mechanical energy pre-storage, controllable locking, and rapid release enables the takeoff system to generate a short-duration high-power output without relying solely on the instantaneous power of the drive motor. In addition, the identified configuration of α = 5° and β = 40° provides a quantitative reference for coordinating the mechanical jumping configuration with the aerodynamic state during the ground-to-air transition. These results provide useful design guidance for the development of compact, reusable, and autonomous takeoff systems for small-scale FWARs.

## Figures and Tables

**Table 1:** Comparison of representative FWARs with jumping mechanisms.

Jump System	Takeoff Strategy	Takeoff Performance	Reusability	Autonomous Capability
Figure 1A Ornibot	Jump-assisted takeoff	/	Reusable	Limited autonomous
Figure 1B Flea beetle-inspired jumping robot	Chemical energy-driven	Jump height: 200 mm	Limited	Limited due to fuel-based
Figure 1C Robo Bee	Piezoelectric actuator	Millimeter-scale flight	Reusable	Limited ground takeoff
Figure 1D Slider-crank ejecting system	Mechanical ejection	Takeoff velocity: 4 m/s	Limited	Limited autonomous
Figure 1E Eagle-scale flapping-wing robot	Leg push-off assisted	Large-scale self-takeoff	Reusable	Autonomous takeoff
Figure 1F Two-wing flap-ping wing MAV	Gear-driven flapping mechanism	/	Reusable	Limited autonomous

**Table 2:** Comparison of the present study with representative biological and bio-inspired jumping studies.

Study	System and Energy-Storage Strategy	Representative Jumping Performance	Aerodynamic Evaluation	Main Focus
Bennet-Clark [26]	*Schistocerca gregaria*; semi-lunar processes and extensor tibiae apodemes	Stored energy: 14 mJ; takeoff velocity: 3.2 m/s; peak acceleration: 180 m/s^2^; jump impulse: 25–30 ms	Not reported	Biological energetics and elastic energy-storage mechanism
Wan et al. [3]	*Locusta migratoria* manilensis; natural hindleg jumping on sand, soil, and wood	Takeoff velocity: 2.3 ± 0.33, 2.3 ± 0.13, and 2.6 ± 0.17 m/s (sand, soil, and wood, respectively)	Not reported	Effect of ground type on locust jumping performance
Mo et al. [6]	*Locusta migratoria*; natural hindleg jumping on substrates with different compliance	Takeoff velocity: 1.87 ± 0.089, 2.07 ± 0.081, and 1.37 ± 0.084 m/s (rigid, compliant, and most compliant substrates)	Not reported	Effect of substrate compliance on takeoff performance
Mo et al. [19]	Locust-inspired six-bar/eight-bar mechanism; torsional-spring energy storage	Jump height: ~0.55 m; distance: ~0.70 m; post-takeoff angular velocity reduced from 8.49 to 6.78 rad/s	Not reported	Takeoff stability and mechanism optimization
Bai et al. [27]	Miniature jumping robot; spring energy storage	Released energy: 600.2 mJ; takeoff velocity: 2.21 m/s; height: 220 mm; distance: 330 mm; energy efficiency: 67%	Not reported	Energy-storage density and jumping performance
This work	*Locusta migratoria*-inspired FWAR; torsion and compression springs with geared transmission and one-way-bearing locking/release	Resultant velocity: 1.955 m/s; height: 168.2 mm; horizontal displacement: 134.6 mm; locking–jumping process: 50 ms	CFD; maximum L/D = 3.005 at α = 5° and β = 40°	Autonomous FWAR takeoff integrating controllable energy storage/release with aerodynamic optimization

## Data Availability

The datasets generated during and/or analyzed during the current study are available from the corresponding authors on reasonable request.

## Abbreviations

The following abbreviations are used in this manuscript:

FWAR	Flapping-Wing Aerial Robots
SLP	Semi-lunar process
TKE	Turbulence kinetic energy
